# Computational Search for Inhibitors of SOD1 Mutant Infectivity as Potential Therapeutics for ALS Disease

**DOI:** 10.3390/ijms26104660

**Published:** 2025-05-13

**Authors:** Marco Carnaroli, Marco Agostino Deriu, Jack Adam Tuszynski

**Affiliations:** 1 DIMEAS, Politecnico di Torino, 10129 Turin, Italy; s305433@studenti.polito.it (M.C.); marco.deriu@polito.it (M.A.D.); 2 Department of Physics, University of Alberta, Edmonton, AB T6G 2E1, Canada

**Keywords:** ALS mutations, molecular dynamics, docking, SOD1 inhibitors

## Abstract

Familial amyotrophic lateral sclerosis (ALS) is a progressive neurodegenerative disease characterized by the selective degeneration of motor neurons. Among the main genetic causes of ALS, over 200 mutations have been identified in the Cu/Zn superoxide dismutase (SOD1) protein, a dimeric metalloenzyme essential for converting superoxides from cellular respiration into less toxic products. Point mutations in SOD1 monomers can induce protein misfolding, which spreads to wild-type monomers through a prion-like mechanism, leading to dysfunctions that contribute to the development of the disease. Understanding the structural and functional differences between the wild-type protein and its mutated variants, as well as developing drugs capable of inhibiting the propagation of misfolding, is crucial for identifying new therapeutic strategies. In this work, seven SOD1 mutations (A4V, G41D, G41S, D76V, G85R, G93A, and I104F) were selected, and three-dimensional models of SOD1 dimers composed of one wild-type monomer and one mutated monomer were generated, along with a control dimer consisting solely of wild-type monomers. Molecular dynamics simulations were conducted to investigate conformational differences between the dimers. Additionally, molecular docking was performed using a library of ligands to identify compounds with high affinity for the mutated dimers. The study reveals some differences in the mutated dimers following molecular dynamics simulations and in the docking of the selected ligands with the various dimers.

## 1. Introduction

The paper reports the results of a computational study on compounds capable of inhibiting the prion-like infection mechanism of superoxide dismutase-1 (SOD1) mutants, which are the basis of amyotrophic lateral sclerosis (ALS). ALS is a severe, progressive neurodegenerative disease characterized by the selective loss of motor neurons in the central nervous system, leading to motor function impairment, paralysis, and eventually death [1]. In its familial form, several genes have been identified as potential causes; the first of these is SOD1, which encodes for the eponymous dimeric protein [2].

Mutations in the SOD1 gene are associated with ALS and may contribute to the disease through various toxic mechanisms [3]. Approximately 200 point mutations have been identified in the SOD1 protein [4], many of which are listed in the ALSOD database [5]. Some mutations, such as G85R, are located near the active site and may interfere with metal ion binding [6]. Others, like A4V, G41D, G41S, and G93A, while distant from the active site, can destabilize the dimer or weaken disulfide bonds, compromising its stability [7].

Initially, it was believed that familial ALS associated with SOD1 was caused by a loss of enzymatic function, leading to cellular toxicity due to the accumulation of free radicals. However, subsequent studies have shown that many mutant variants retain high enzymatic activity, and SOD1’s antioxidant function can be compensated by other proteins of the same family [8,9]. Therefore, it has been hypothesized that mutations cause a toxic gain of function.

Mutations can render metal ion binding ineffective: if the enzyme no longer binds zinc, the copper required for the dismutation reaction will also fail to bind properly, leading to reversed enzymatic activity and excessive production of superoxide ions [10]. Moreover, the release of metal ions may promote neurotoxicity [11]. Another effect of mutations is protein destabilization, which can cause SOD1 to lose its native conformation and aggregate [12,13].

A key mechanism in ALS is the prion-like conversion of mutant SOD1: misfolded proteins can induce the misfolding of native proteins and spread between cells [14,15]. This process can occur through two mechanisms. The first is template-directed misfolding, where a misfolded monomer induces a native monomer to lose its correct structure [16,17]. This happens through the exposure of hydrophobic surfaces, recognition and binding to the wild-type monomer, and subsequent misfolding propagation.

The second mechanism is nucleated polymerization, where small aggregates of mutant SOD1 act as “seeds” for the formation of amyloid fibrils [18]. Initially, these aggregates form slowly, but once stabilized, they can interact with wild-type proteins and induce their conversion into pathological forms. Fibrils can fragment and generate new nuclei, accelerating disease spread among cells [19].

Both mechanisms contribute to ALS progression by increasing SOD1’s propensity to expose hydrophobic surfaces and promoting the formation of toxic aggregates [20,21].

It is hypothesized that in its mutated form, this protein may destabilize and subsequently induce the destabilization of the wild-type protein through a prion-like mechanism. Although native SOD1 consists of two identical monomers, the presence of a mutant monomer may alter the structural balance. Following a mechanism similar to that of prions, mutant monomers would transfer their conformational characteristics to wild-type monomers they come into contact with, inducing them to adopt an incorrect, destabilizing conformation. The resulting dimer, composed of a mutant monomer and an “infected” wild-type monomer, would dissociate, allowing the mutant monomer to propagate the misfolding of the protein without the intervention of external agents, just like a prion [22].

The study reported here was divided into three phases. In the first phase, eight monomeric structures in PDB format were obtained for further analysis. The first structure represented the wild-type (wt) form, while each of the remaining structures contained a point mutation (A4V, G41D, G41S, D76V, G85R, G93A, and I104F). As these mutated structures were not available from crystallography, they were predicted using AlphaFold2 (https://alphafold.ebi.ac.uk/ (accessed on 10 May 2024)). However, the resulting predicted structures lacked metal ions, which are essential for enzymatic activity and, above all, protein stability. Therefore, the missing ions were added manually. The monomers were then combined using the Haddock software (https://rascar.science.uu.nl/haddock2.4/ (accessed on 15 May 2024)), resulting in eight dimers, including the completely wild-type protein, namely: wt+wt, wt+A4V, wt+G41D, wt+G41S, wt+D76V, wt+G85R, wt+G93A, and wt+I104, which were studied experimentally in [23].

In the second phase, after careful preparation, 1200 ns MD simulations were performed using the Amber software (https://ambermd.org/ (accessed on 30 May 2024)) on the obtained structures. Following the simulations, a series of analyses and conformational clustering were carried out to highlight the differences between the mutated and wild-type structures.

In the third phase, 35 ligands from the Drugbank database of approved investigational drugs (https://go.drugbank.com/ (accessed on 5 August 2024)) were selected and, using the AUTODOCK VINA software (https://vina.scripps.edu/ (accessed on 10 September 2024)), a blind docking (since no information on the protein’s specific binding site was available) was conducted for each ligand on every structure, both mutated and not, for a certain number of iterations. This allowed data collection on binding affinity for each ligand–protein pair. Finally, clustering was performed on the poses obtained for each ligand to determine if it tended to bind recurrently to a specific protein region, thereby providing insights into the ligand’s specificity.

## 2. Results

### 2.1. Structure of the Human Wild-Type Holo Protein

**Figure 1:**
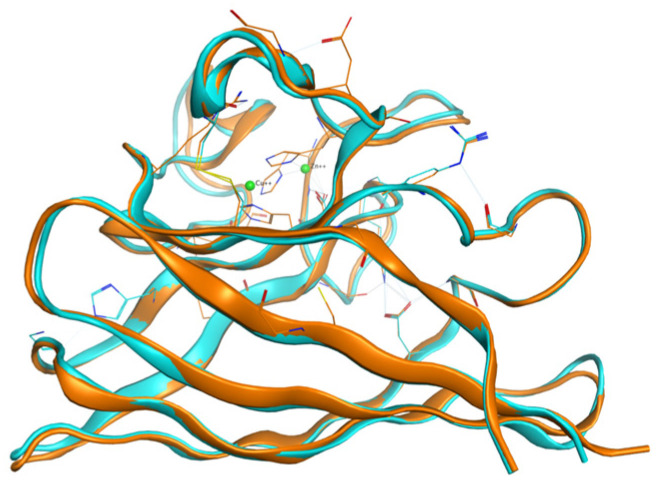
The monomer derived from PDB entry “3ecu” is shown in light blue, superimposed on the monomer derived from “7wwt” in orange, with the metal ions depicted in green.

The monomeric human SOD1 structure was derived from the Protein Data Bank (https://www.rcsb.org/) with the entry ID: “3ecu” (chain A) and the animal one derived from PDB as the “7wwt” (chain A) exhibit, and after alignment of the amino acid sequences, a sequence identity of 81.7% and sequence similarity of 88.8% was found. Additionally, the RMSD of the two structures after superposition (Figure 1) is 0.526 Å. The fact that the structures are so similar implies that the location of metal ions in the animal protein can be used, after the superposition, to locate the same ions in the human structure.

### 2.2. Mutant Monomer Generation

#### 2.2.1. Mutated Sequences

The output file from the MATLAB R2023b code, containing the monomer sequences in FASTA format, is available below. The point mutations are highlighted in yellow in each sequence in Table 1.

#### 2.2.2. Mutated Structures

**Figure 2:**
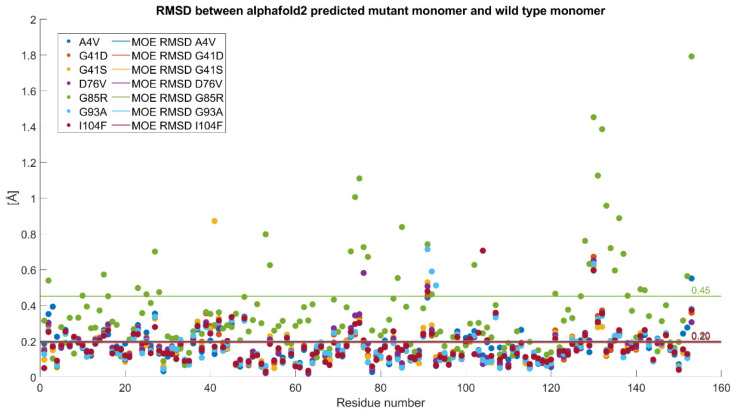
Comparison between the RMSD of the various mutants (shown as dots in the legend) based on their positions in the amino acid sequence. The horizontal lines, following the same color scheme, represent the RMSD values calculated for each mutant.

The mutated structures predicted with AlphaFold2 were superimposed onto the wild-type structure, and the RMSD was calculated for each combination. This is visualized in Figure 2 both for each residue and as a single value (according to the MOE RMSD definition, see Section 4). The maximum RMSD thus calculated (related to the G85R mutant) is 0.4511 Å, indicating that the mutant PDB structures do not show significant differences compared to the wild-type structure, since we only considered point mutations. However, the subsequent MD simulations performed on the dimers are intended to determine whether these mutations may lead to substantial conformational differences.

### 2.3. Generation of Dimers

#### Dimer Interface Characteristics

**Figure 3:**
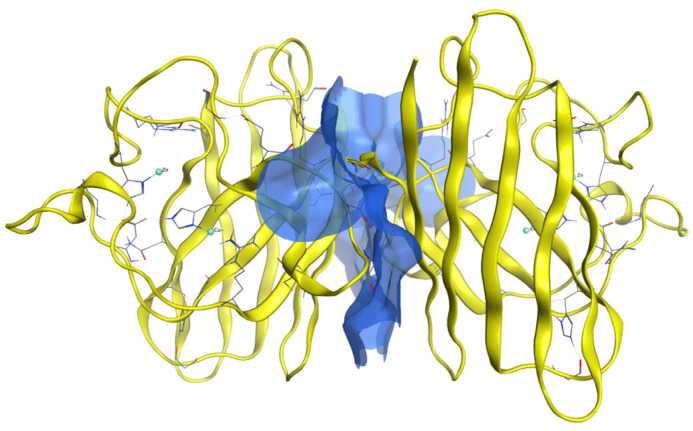
A generic SOD1 dimer shown in yellow with the surface which includes the contacts between the two monomers highlighted in blue.

The dimer generation was performed using the protein–protein docking software Haddock. Dimerization occurs primarily due to hydrogen bonds, leading to a more complex, and in the case of SOD1, more functionally efficient and stable dimer structure (Figure 3).

**Figure 4:**
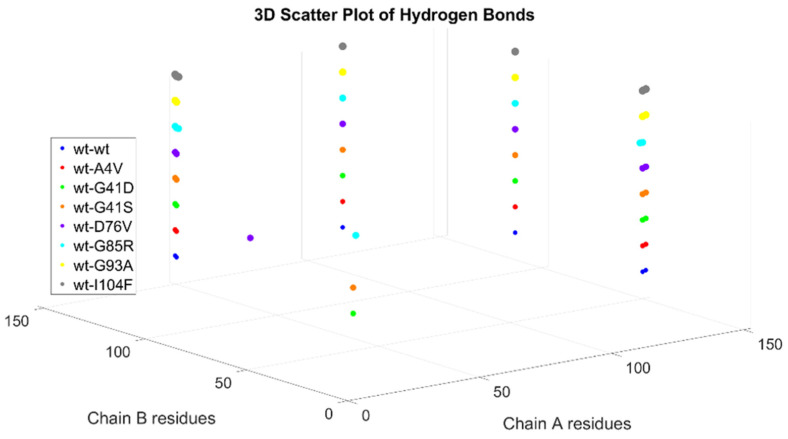
Illustration of the pairs of residues from chain A and chain B that are involved in the hydrogen bonds at the interface of the various dimers.

The eight generated dimers share the same behavior during the dimerization process; hence, Figure 3 is representative of all the dimers. The residues implicated in the dimerization process are 50–53, 114, 148, and 150–153 [24]. Figure 4 illustrates for each dimer, after the protein–protein docking process, which residues are implicated in the hydrogen bonds between chain A (first monomer) and chain B (second monomer). It is clear that most of the dimers share the same hydrogen bonds, even though dimers wt+G41D, wt+G41S, and wt+D76V have another hydrogen bond, and the latter, in a different position.

### 2.4. MD Simulations: Data Analysis

#### 2.4.1. RMSD

**Figure 5:**
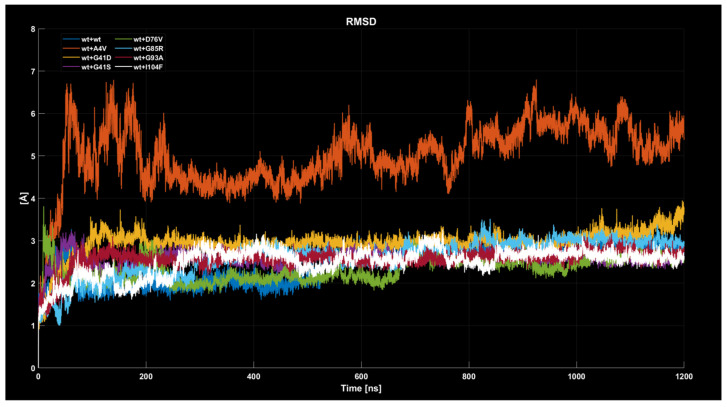
Time evolution of RMSD values for all eight dimers analyzed.

In the case of the wild-type dimer (wt+wt dimer, see Figure 5), the RMSD exhibits a rapid initial increase during the first 50 ns. Over the next 50 ns, the value stabilizes around 2 Å, reflecting a relatively compact and stable conformation. Between 100 and 600 ns, the RMSD remains constant with minimal fluctuations around 2 Å. However, after 600 ns, a gradual increase in RMSD is observed, converging at approximately 3 Å, suggesting potential structural reorganization or larger-scale fluctuations in the latter half of the simulation. Despite this, the amplitude of oscillations in the final phase remains limited, indicating that the dimer retains overall stability, albeit with slightly increased global flexibility. For the wt+A4V dimer, the RMSD rises sharply during the first 100 ns, reaching approximately 4 Å. Beyond this point, the trajectory exhibits highly oscillatory behavior, with values fluctuating between 4 and 6 Å throughout the simulation. These fluctuations reflect significant and continuous structural changes, with no convergence toward a stable configuration. The larger oscillation amplitude and higher average RMSD compared to the other dimers indicate increased structural instability, which persists during the entire simulation. The wt+G41D dimer displays an initial rapid increase in RMSD to 3 Å within the first 100 ns. Following this equilibration phase, the value stabilizes, showing minimal oscillations for the remainder of the simulation. The plateau around 3 Å suggests that the system achieves a stable configuration. The limited fluctuations after stabilization indicate a structure that, while not converging to a lower RMSD value, maintains consistent behavior over time. For the wt+G41S dimer, the RMSD curve shows a rapid increase within the first 50 ns, followed by a gradual stabilization around 2.5 Å after approximately 100 ns. Fluctuations remain minimal throughout the simulation, indicating that the system reaches a stable configuration relatively early. The wt+D76V dimer exhibits an initial increase in RMSD during the first 50 ns, with some oscillations not as pronounced as for the wt+A4V dimer, followed by stabilization around 2.5 Å. For the wt+G85R dimer, the RMSD behavior is characterized by a slow increase with moderate fluctuations before stabilizing around 3 Å. In the case of the wt+G93A dimer, the RMSD curve shows a sharp initial increase, followed by stabilization around 3 Å after the first 100 ns. This system, like wt+G41S, demonstrates exceptional stability, with extremely low fluctuations and a nearly flat trajectory throughout the simulation. This could indicate that the G93A mutation does not introduce significant changes to the structural stability of the dimer compared to the wild type. Finally, the wt+I104F dimer displays behavior similar to that of the wt+G93A dimer, with an initial increase in RMSD followed by stabilization around 3 Å, but with some faster fluctuations. The fluctuations remain minimal, and the RMSD trajectory remains stable until the end of the simulation. In summary, the detailed analysis of the RMSD curves highlights greater structural instability for the wt+A4V dimer, while the other dimers exhibit relatively similar behaviors, stabilizing between 2.5 and 4 Å with limited fluctuations after the first 100 ns.

#### 2.4.2. Radius of Gyration

**Figure 6:**
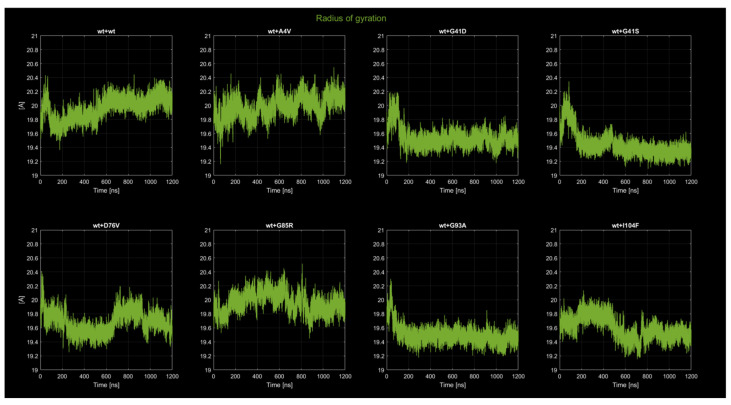
Time evolution of the radius of gyration for all eight dimers.

The analysis of the radius of gyration (Rg, see Figure 6) reveals distinct trends for each dimer, providing insights into the structural compactness throughout the simulation. In the wt+wt dimer, the Rg initially fluctuates between 19.6 Å and 20.0 Å for the first 100 ns, indicating a relatively compact structure with moderate flexibility. As the simulation progresses, fluctuations slightly increase, ranging between 19.5 Å and 20.2 Å, but without significant shifts in the mean value. This suggests that the wt+wt dimer maintains a stable and equilibrated structure with no major rearrangements or loss of compactness over the 1200 ns. For the wt+A4V dimer, the Rg consistently fluctuates between 19.2 Å and 20.5 Å, reflecting higher flexibility and a wider fluctuation range compared to the wt+wt system. The persistence of these fluctuations throughout the simulation indicates that the A4V mutation prevents the dimer from achieving a tightly packed equilibrium, suggesting a destabilizing effect on the protein’s compactness and a greater degree of structural rearrangement. The wt+G41D dimer exhibits an initial stabilization phase where the Rg increases from approximately 19.4 to 20.00 and decreases from 19.8 Å to 19.4 Å within the first 100 ns, followed by minimal fluctuations for the remainder of the simulation. This behavior suggests that the G41D mutation promotes a more compact and stable conformation after an early equilibration period. Similarly, the wt+G41S dimer shows an increase followed by a sharp decrease in Rg from 20.0 Å to 19.4 Å within the first 50 ns, followed by stable values with negligible fluctuations around 19.4 Å. The data indicate that the G41S mutation allows the dimer to quickly achieve and maintain a tightly packed structure, resembling the behavior observed for the G41D mutant. In the wt+D76V dimer, the Rg oscillates significantly between 19.6 Å and 20.2 Å throughout the entire simulation, reflecting a dynamic equilibrium between compact and expanded states. This pronounced fluctuation pattern suggests that the D76V mutation introduces a destabilizing effect, preventing the dimer from achieving a consistently compact configuration and possibly promoting conformational changes. The wt+G85R dimer shows fluctuations between 19.6 Å and 20.2 Å, similar to the wt+D76V system, but the range of oscillations is slightly narrower, indicating a relatively more stable structure. Despite this, the G85R mutation still introduces noticeable flexibility compared to the wt+wt, wt+G41D, and wt+G41S systems. The wt+G93A dimer exhibits one of the most stable Rg profiles, with fluctuations limited to a range of 19.6 Å to 19.8 Å after an initial equilibration phase. This suggests that the G93A mutation does not significantly impact the compactness or stability of the dimer, which remains tightly packed throughout the simulation. The wt+I104F dimer displays similar fluctuations to the wt+D76V dimer. Overall, the wt+wt, wt+G41D, wt+G41S, and wt+G93A dimers exhibit stable Rg profiles with tightly packed conformations, whereas the wt+A4V, wt+D76V, wt+G85R, and wt+I104F dimers show broader fluctuations and increased flexibility, reflecting varying degrees of destabilization and reduced compactness induced by the respective mutations. These observations highlight the differential impact of mutations on the structural dynamics of the dimers, with trends in compactness and stability observed across the set of simulations.

#### 2.4.3. Solvent-Accessible Surface Area (SASA)

**Figure 7:**
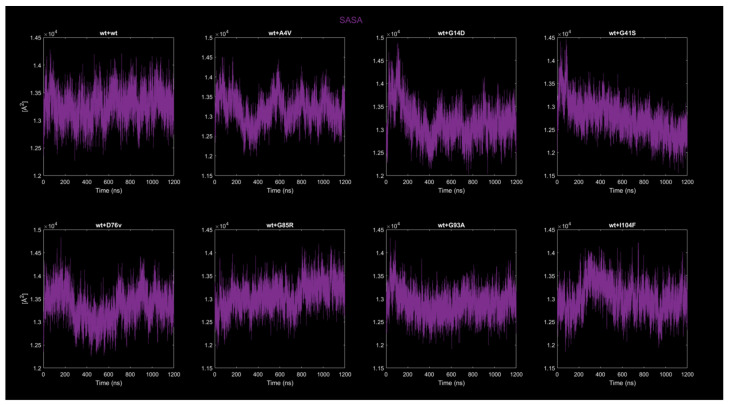
Time evolution of SASA for all eight dimers.

Figure 7 shows the SASA as a function of time for the eight different dimers of the SOD1 protein. The vertical axis represents the SASA in Å^2^, and the horizontal axis represents time in nanoseconds. For the wt+wt dimer, the SASA values exhibit consistent oscillations within the range of 1.25 × 10^4^ Å^2^ and 1.4 × 10^4^ Å^2^ throughout the simulation. The fluctuations are relatively uniform, with no abrupt spikes or drops observed. There is no discernible trend of increase or decrease in SASA values over time, and the oscillations appear to occur at regular intervals. For the wt+A4V dimer, the SASA values fluctuate between 1.25 × 10^4^ Å^2^ and 1.45 × 10^4^ Å^2^, showing broader oscillations compared to wt+wt. The fluctuations are less regular, with periods of higher variability interspersed with intervals of relatively smoother dynamics. No clear overall trend is observed, but the irregularity in oscillations suggests transient structural dynamics during the simulation. For the wt+G41D dimer, the SASA curve starts at a maximum of approximately 1.45 × 10^4^ Å^2^ and decreases progressively to about 1.25 × 10^4^ Å^2^ over the first 400 ns. The decrease is relatively smooth and monotonic, without large spikes or oscillations during the downward trend. After 400 ns, the SASA stabilizes around 1.3 × 10^4^ Å^2^ and exhibits small, regular fluctuations around this value until the end of the simulation. For the wt+G41S dimer, the SASA begins at a value of approximately 1.35 × 10^4^ Å^2^ and gradually decreases to around 1.2 × 10^4^ Å^2^ by the end of the simulation. The decrease is less steep than in wt+G41D, but constant over the course of the simulation. By the final stages of the simulation, the SASA stabilizes at around 1.2 × 10^4^ Å^2^, indicating a consistent end-state value. For the wt+D76V dimer, the SASA values consistently oscillate between 1.25 × 10^4^ Å^2^ and 1.4 × 10^4^ Å with a behavior between the wt+wt dimer and wt+A4V dimer. For the wt+G85R dimer, the SASA values exhibit an increase, opposite to the wt+G41S dimer but with fluctuations similar to the wt+wt dimer. For the wt+G93A dimer, the SASA shares a similar behavior to the wt+wt dimer, except for fluctuations with lower values. For the wt+I104F dimer, the SASA curves are characterized by some initial fluctuations that exhibit a wide amplitude, with sporadic peaks and troughs within this range. After 600 ns, the amplitude of fluctuations decreases, and the SASA stabilizes around a narrower range between 1.25 × 10^4^ Å^2^ and 1.35 × 10^4^ Å^2^. In general, the wt + wt dimer is stable with regular fluctuations, while mutants show more dynamic behavior: some exhibit progressive SASA decreases (e.g., wt+G41D and wt+G41S), others show increases (e.g., wt+G85R) or irregular oscillations (e.g., wt+A4V and wt+I104F). These changes reflect structural adaptations specific to the mutations.

#### 2.4.4. RMSF

**Figure 8:**
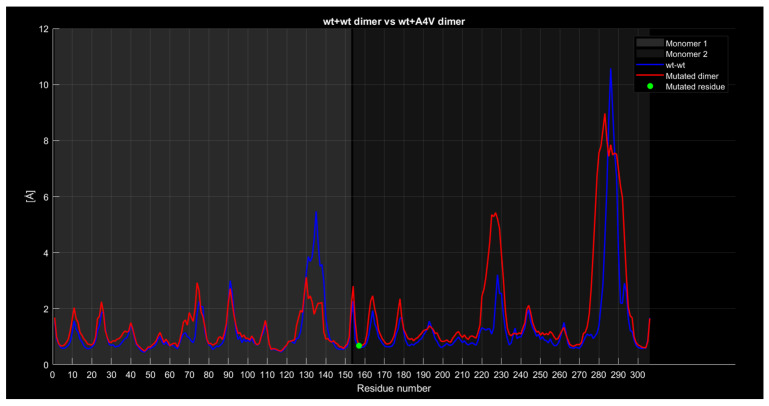
The wt+A4V dimer RMSF compared to the wild-type dimer.

The following graphs illustrate RMSF comparisons between the wild-type dimers and mutant dimers. The horizontal axis represents the residue number of the protein, while the vertical axis indicates the RMSF values in angstroms, measuring the average flexibility of each residue during the simulation. Each graph overlays two main curves. The blue curve represents the wild-type (wt+wt) dimer RMSF, while the red curve represents the mutated dimer RMSF. Additional details in the graphs include a green dot, which marks the exact location of the mutated residue along the protein sequence, and a legend distinguishing between the two monomers forming the dimer (Monomer 1 and Monomer 2). These graphs enable direct comparisons of residue flexibility between wild-type and mutated dimers, highlighting significant differences in key regions. The RMSF trend comparison between the wild-type wild-type dimer and the wild-type A4V dimer (Figure 8) shows some differences despite a generally similar trend. Specifically, the red curve, representing the mutated dimer, displays lower values in the residue range from 130 to 140, higher values around residue 225 with a difference of approximately 2 angstroms, and a less pronounced peak around residue 285 with a difference of about 1 angstrom.

**Figure 9:**
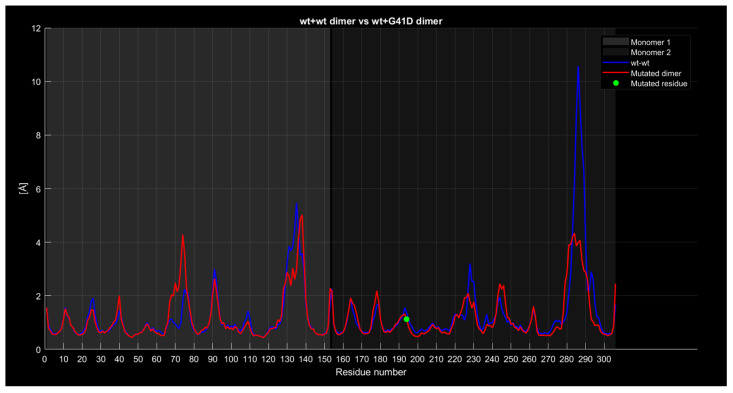
The wt+G41D dimer’s RMSF compared to the wild-type dimer.

Similarly, the comparison between the wild-type wild-type dimer and the wild-type G41D dimer (Figure 9) reveals a similar trend overall, but significant differences are observed around residue 230 and, notably, around residue 270, where the red curve has a peak 6 angstroms lower than the blue one.

**Figure 10:**
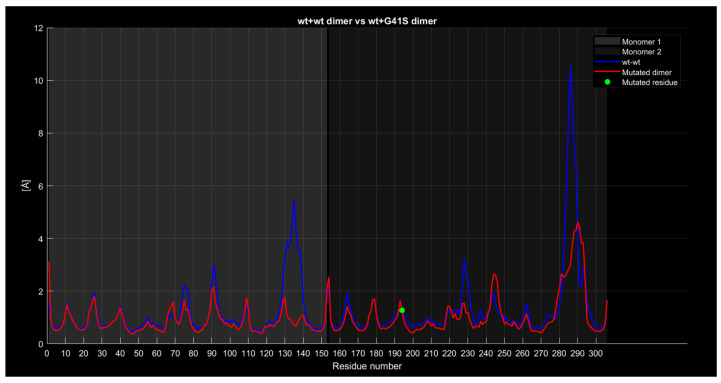
The wt+G41S dimer RMSF compared to the wild-type dimer.

For the wild-type wild-type dimer and the wild-type G41S dimer (Figure 10), while the general trend remains consistent, the blue curve exhibits a 4-angstrom lower value around residue 135, a 2-angstrom lower value around residue 230, and a 5-angstrom lower value around residue 285.

**Figure 11:**
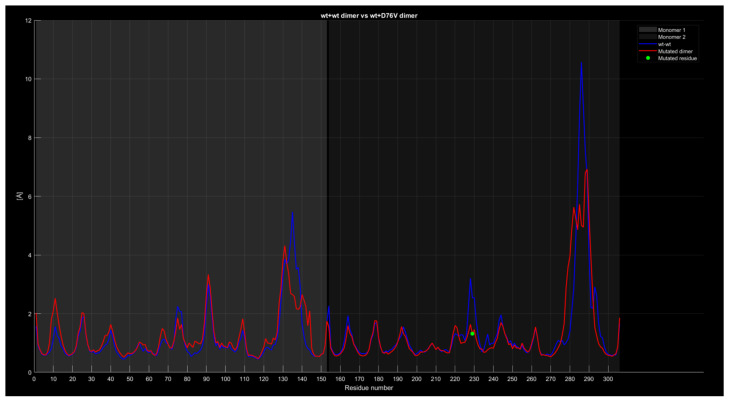
The wt+D76V dimer RMSF compared to the wild-type dimer.

In the case of the wt+wt dimer and the wt+D76V dimer (Figure 11), the red curve shows approximately 1 angstrom lower values around residue 135, 2 angstroms lower around residue 230, and about 3 angstroms lower around residue 285 compared to the blue curve. At first glance, the mutation of residue 76 in the second monomer, corresponding to residue 229 of the entire protein and marked by a green dot, appears to contribute to the variation in the second peak of the red curve compared to the blue one. However, given that this behavior and difference are common across other graphs comparing proteins with different mutations, there is no definitive correlation between the mutation’s position and the observed differences between the curves.

**Figure 12:**
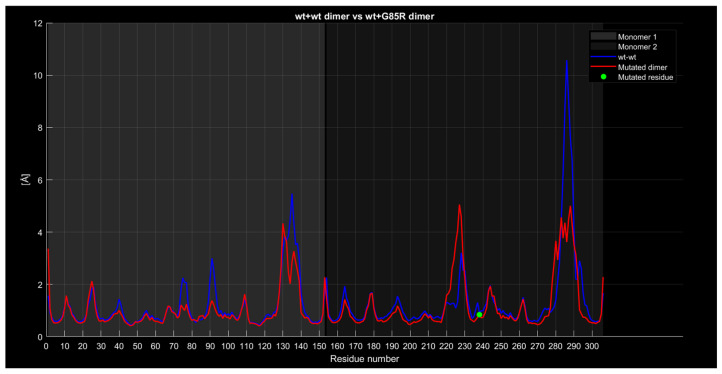
The wt+G85R dimer’s RMSF compared to the wt+wt dimer.

For the wt+wt dimer and the wt+G85R dimer (Figure 12), the red curve generally exhibits similar characteristics to the previous cases, with lower values around residues 135 and 285. However, around residue 225, the red curve exceeds the blue one by approximately 2 angstroms.

**Figure 13:**
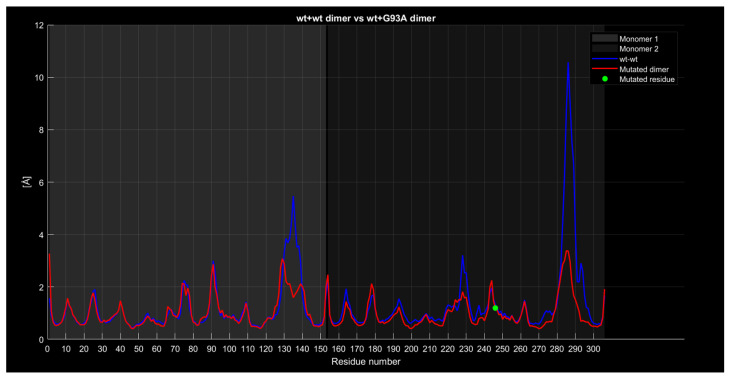
The wt+G93A dimer’s RMSF compared to the wt+wt dimer.

The wt+wt dimer and the wt+G93A dimer comparison (Figure 13) shows the red curve exhibiting similar characteristics to the previous cases, with lower values around residues 135, 225, and 285.

**Figure 14:**
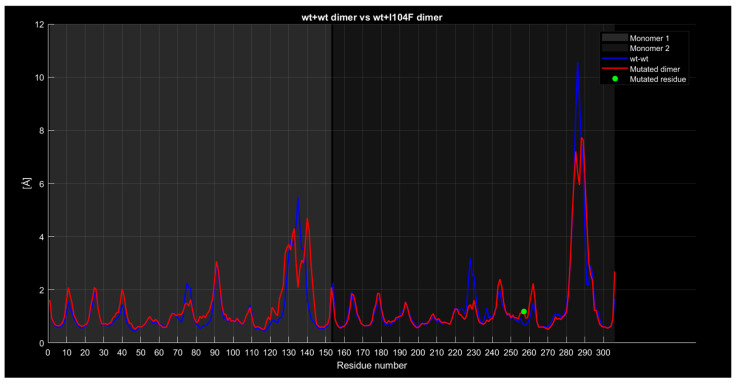
The wt+I104F dimer’s RMSF compared to the wt+wt dimer.

Similarly, for the wt+wt dimer and the wt+I104F dimer (Figure 14), the red curve displays similar characteristics, with lower values around residues 135, 225, and 285.

**Figure 15:**
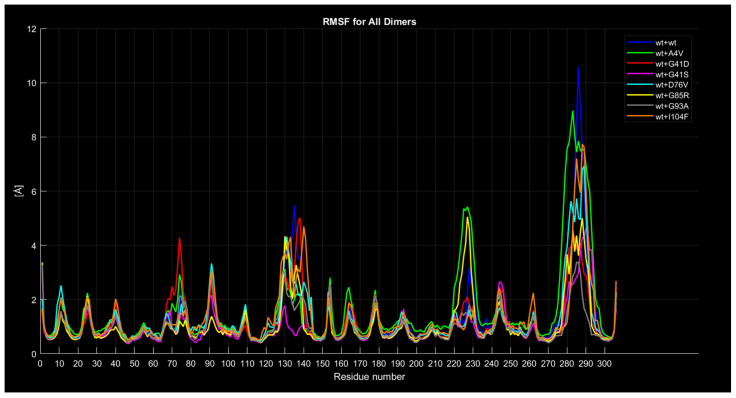
Mutant RMSF curves compared to the wild type curve.

In conclusion, all dimers with mutations tend to show generally lower RMSF values around the previously mentioned residues and hence, lower amplitudes of spikes in those locations. However, dimers with A4V and G85R mutations in the second monomer stand out, displaying a higher peak compared to the completely wild-type dimer around residue 225. Figure 15 is a summary image with all the RMSF curves superimposed for clarity.

**Figure 16:**
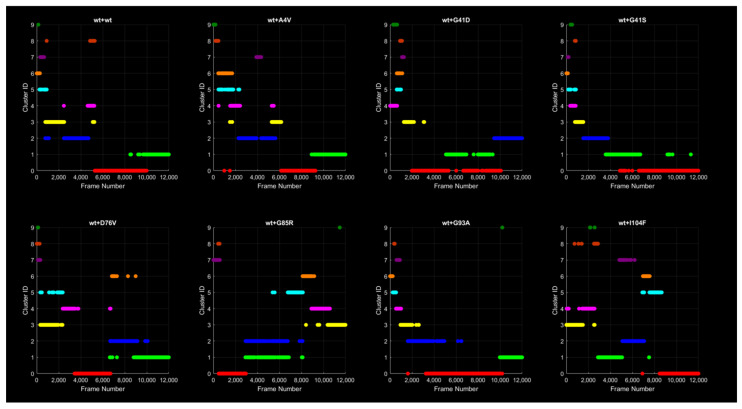
Evolution of cluster population over time. Each trajectory frame is assigned to a specific cluster, represented by a distinct color. Note that although the same color scheme is used, a cluster of a given color in one protein does not correspond to the cluster of the same color in another protein.

Figure 16 shows the clusters to which the frames of the trajectory from each MD simulation belong for each dimer. It can be observed that after conformational clustering, 10 clusters were found for each protein. As the number of frames increases during the simulation, the frames transition into different conformations, and thus different clusters. The clusters are numbered from 0 to 9, ranked from the most to the least populous. Although the red cluster identified as 0 is the largest, possibly containing the most frames and therefore being the most recurring and representative of the protein structure, it is not the final cluster in all the dimers, i.e., the one containing the last frames of the protein. In fact, there is some variability among the dimers regarding the distribution of frames across the different clusters. However, it is difficult to attribute this variability to the mutation. Additionally, the k-means algorithm was applied, which requires prior knowledge of the number of clusters, set to 10 in this case. A clustering algorithm that does not require prior knowledge of the number of clusters can be applied in future studies to verify whether the dimers adopt a larger number of conformations compared to the wild type.

**Figure 17:**
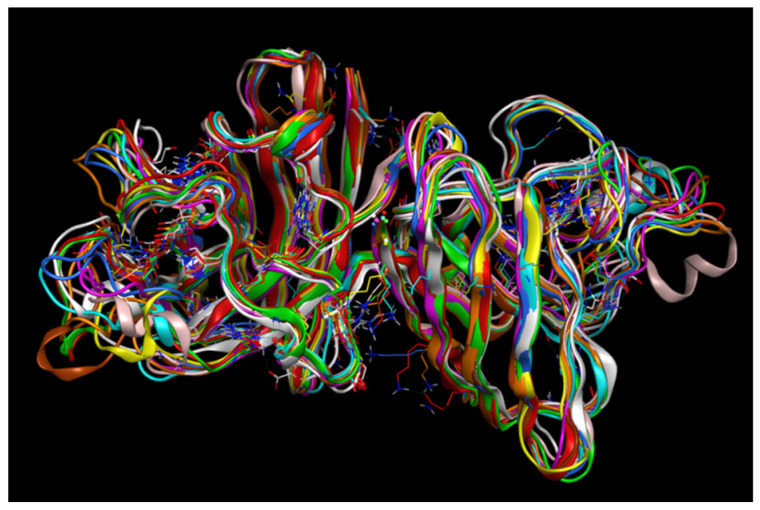
Superposition of the wt+wt dimer’s representative structures, with each color corresponding to a specific cluster.

In Figure 17, the structures corresponding to the various clusters of the wt+wt dimer are shown, colored differently compared to the previously presented graph, and overlaid. Note that the orange structure representing cluster 5 and the pale pink structure corresponding to cluster 7 display alpha helices that deviate from those in other structures, indicating the particular conformations this type of protein can adopt.

#### 2.4.5. Ramachandran Plots

**Figure 18:**
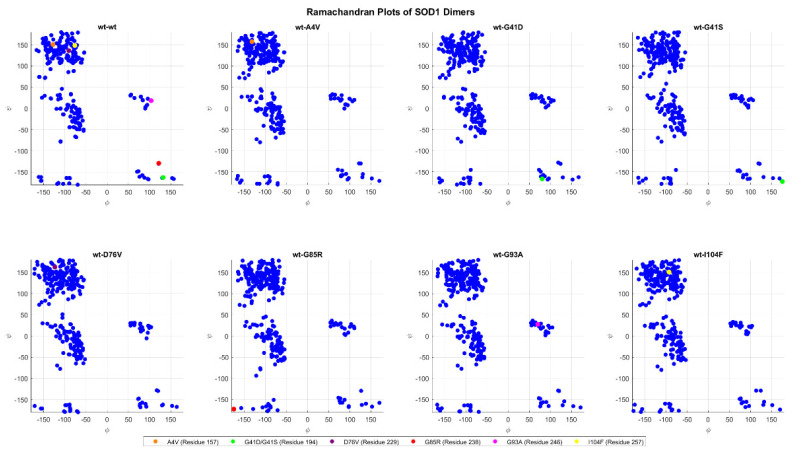
Ramachandran plot of protein residues for all the dimers. Each blue point represents the pair of φ and ψ values for a specific generic residue, while the colored points represent the same but for a mutated residue.

Figure 18 shows the Ramachandran plots comparing the wt+wt dimer with the mutants, highlighting the variations in the torsional angles phi (*ϕ*) and psi (*ψ*) of the mutated residues. The structures used are the ones generated via AlphaFold and Haddock before the MD simulations. In the comparison with wt+G41D, the green dot of residue A4 shifts significantly from (131, −162) to (80, −166), indicating a major conformational change, especially in the phi angle. Similarly, in wt+G41S, the same residue A4 shifts even more drastically from (131, −162) to (175, −172), suggesting that both mutations G41D and G41S cause significant changes in the torsional angles, with important conformational effects but in different directions. In the case of wt+G85R, the red dot of residue G85 shows a marked shift from (121, −129) to (−174, −172), with a complete reversal of the phi angle and a large variation in the psi angle, indicating a notable perturbation in the local structure. These three mutations, G41D, G41S, and G85R exhibit the most evident variations in the comparisons between the wild type and mutated.

**Figure 19:**
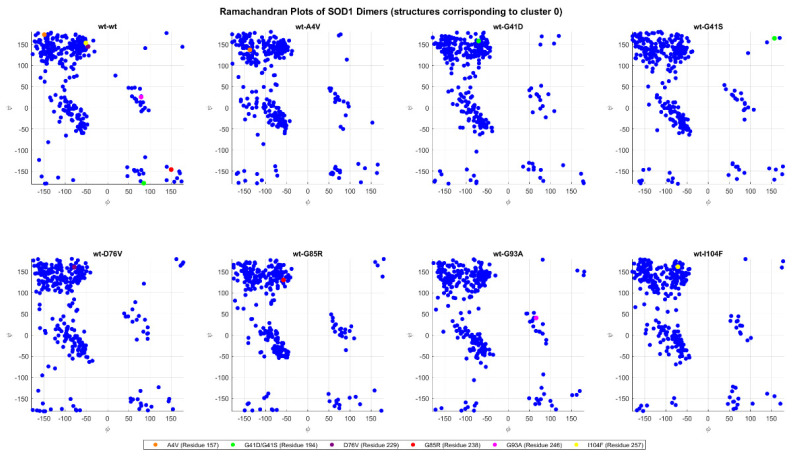
The same as in Figure 18, but in this case, the dimer structures are derived from cluster 0 after the conformational clustering.

Figure 19 shows similar plots to those of Figure 18, but for the structures resulting from the conformational clustering. For each dimer, they represent the structural conformation in cluster 0, the most sizeable cluster.

The mutated forms, whose mutation is represented by the colored dots show differences in torsional angles compared to the wt+wt form. For example, in the wt+G41D mutation (green dot), the mutated residue has coordinates (−70, 158) compared to (85, −177) in the wt+wt, indicating a significant change that could destabilize the local structure. Similarly, in the wt+G41S mutation (another green dot), large differences are observed, with coordinates (156, 164) compared to (85, −177) in the wt+wt, highlighting a significant alteration in angular conformation. Another mutation with a strong impact is wt+G85R (red dot), with coordinates (−57, 130) compared to (150, −145) in the wt+wt, suggesting a relevant change in the local secondary structure. In the wt+A4V mutation (orange dot), the coordinates (−136, 137) compared to (−149, 172) in the wt+wt show a variation in ϕ with a slight difference in ψ, potentially affecting the orientation of the structure.

### 2.5. Docking Results

#### Binding Energies and Spatial Pose Clustering Results

With respect to the wt+wt dimer, ligands 22, 23, 38, 36, and 28 exhibit the highest affinity, while ligands 8, 24, 37, 13, and 34 tend to form the largest clusters. A similar pattern is observed in the wt+A4V dimer, where ligands 22, 36, 38, 23, and 29 perform the best, and ligand 24 stands out for forming the largest cluster. For both the wt+G41D and wt+G41S dimers, ligands 22, 23, 36, 38, and 28 consistently show the highest affinity, with ligands 6, 34, and 37 forming the largest clusters. In the case of the wt+D76V dimer, ligands 36, 22, 38, 23, and 29 perform the best, while ligands 16, 34, and 37 show a strong tendency to form large clusters. In the wt+G85R dimer case, ligands 22, 38, 23, 36, and 28 again demonstrate top performance, with ligands 6,13, and 37 forming the largest clusters. For the wt+G93A dimer, ligands 22, 38, 36, 29, and 23 are the top performers, while ligands 3, 20, 24, and 36 form the largest clusters. Finally, for the wt+I104F dimer, ligands 22, 36, 29, 38, and 23 maintain high performance, with ligands 24 and 34 forming the most prominent clusters. The graphics explaining those results can be found in Supplementary Information. Overall, VINA docking scores highlight ligands 22, 36, 38, and 23 as having the highest affinity for their target structures. However, ligand 23, rotenone, is a toxic pesticide harmful to humans, and even though it shares over 60% structural similarity (computed using the DrugBank tool) with ligand 22, which has been studied for ALS treatment, it is not included in the list.

**Figure 20:**
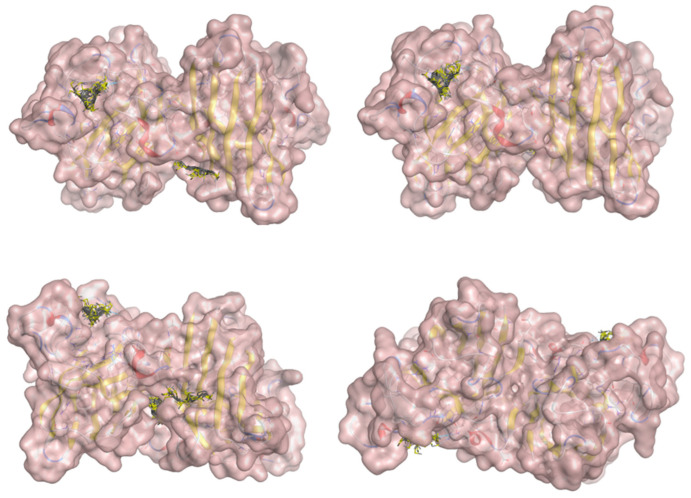
Three-dimensional representation of dimer wt+wt in pink with ligand 22 docked multiple (100) times.

**Figure 21:**
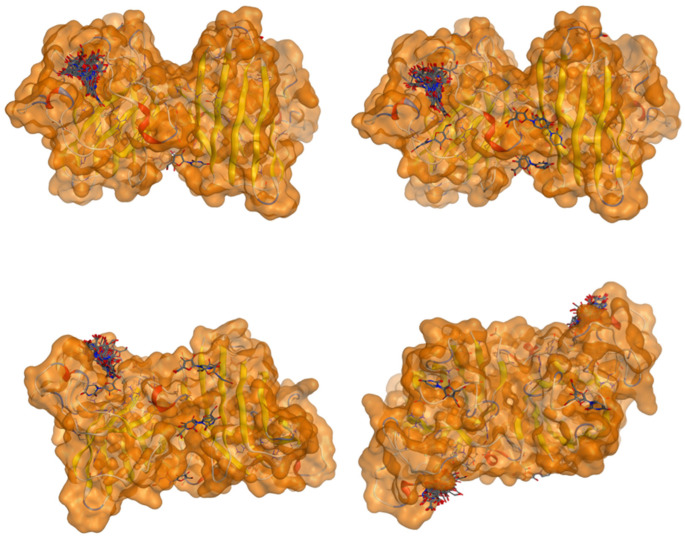
Three-dimensional representation of dimer wt+wt in orange with ligand 36 docked multiple (100) times.

**Figure 22:**
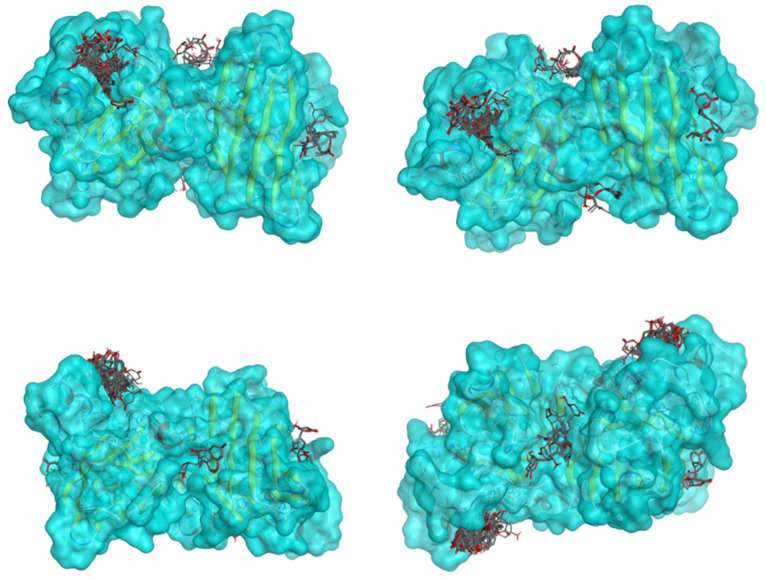
Three-dimensional representation of dimer wt+wt in light blue with ligand 38 docked multiple (100) times.

All 100 poses for each of the top ligands can be visualized in 3D together with the wt+wt dimer in Figure 20, Figure 21 and Figure 22 from different perspectives (front, back, bottom, and top). Preferred ligand-binding sites can already be observed as concentrated clusters of ligands.

**Figure 23:**
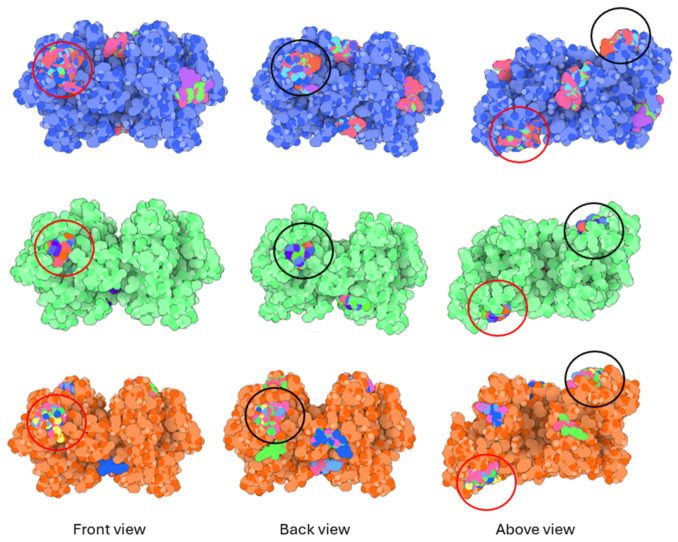
Graphical representation of ligands 37, 24, and 34 bonded to the wt+wt dimer. Each line represents the wt+wt dimer bonded 100 times to a specific ligand in three perspective views. The two binding regions shared between them are circled in red and black.

Regarding the ligands that form the largest clusters as a result of the pose clustering algorithm—namely ligands 37, 24, and 34—it is evident that they all consistently bind to at least two regions of the wt+wt protein, specifically one in each monomer of the dimer. These regions are highlighted in red and black, respectively, in Figure 23. However, there are also other regions located at the interface between the two monomers in the lower part of the dimer. Notably, the regions where these ligands tend to concentrate correspond to those occupied by the previously mentioned high-affinity ligands—namely ligands 22, 36, and 38—visible in Figure 20, Figure 21 and Figure 22.

For ligands 22, 36, and 38, the top-ranked poses were selected based on the Vina scoring function. Specifically, these correspond to the best-scoring model (model 1) from each of the 10 independent docking runs and are reported in Table 2, Table 3 and Table 4.

A detailed overview of the 100 interactions between the ligands and the wt+wt dimer is provided in Table 5, Table 6 and Table 7. The models predicting ligand binding at the dimer interface—i.e., involving both monomers (chain A and chain B)—are highlighted in blue. Specifically, interface binding is observed in 10 models for ligand 22, 4 for ligand 36, and 11 for ligand 38. So from the data, it seems that ligands 22 and 38 tend to bind more at the interface than ligand 36.

## 3. Discussion

In this work, seven SOD1 mutations (A4V, G41D, G41S, D76V, G85R, and I104F) were selected, and three-dimensional models of SOD1 dimers composed of one wild-type monomer and one mutated monomer were generated, along with a control dimer consisting solely of wild-type monomers. Molecular dynamics simulations were conducted to investigate conformational differences between the dimers. Additionally, molecular docking was performed using a library of ligands to identify compounds with high affinity for the mutated dimers. The study reveals some differences in the mutated dimers following molecular dynamics simulations and in the docking of the selected ligands.

Future studies could benefit from exploring a larger pool of compounds and analyzing more mutations, given the over 200 affecting the SOD1 gene and protein. Increasing docking poses would enhance binding region consistency and aid in identifying binding sites. Additionally, molecular dynamics simulations of dimer–ligand complexes could assess potential ligand effects on protein stability and conformational variability.

## 4. Materials and Methods

The computational workflow consisted of three phases.

In phase 1, the wild-type holo SOD1 protein was obtained. The PDB file ‘3ecu’, containing two identical SOD1 dimers but lacking metal ions, was downloaded from the RCSB PDB database. To restore the metal ions essential for dimer stability, the SOD1 structure from ‘7wwt’, which includes these ions in the correct crystallographic positions, was used. Using the MOE software (https://www.chemcomp.com/en/Products.htm (accessed on 10 May 2024)) [25], only the first monomer (chain A) was retained from each structure. The sequences were aligned to assess similarity and identity, and the monomers were superimposed to calculate RMSD. The 7wwt monomer was then removed, leaving the 3ecu monomer with integrated metal ions.

To generate mutated structures, seven mutant sequences (A4V, G41D, G41S, D76V, G85R, G93A, and I104F, selected based on [23]) were created by modifying the wild-type sequence obtained from UniProt. A MATLAB script was developed to substitute specific residues within a FASTA file according to specifications in a text file [26]. The resulting sequences were processed with AlphaFold2 via a Python script on Google Colab [27] using “3ecu” (chain A) as a template. Each mutant structure was then stabilized by adding zinc and copper ions following the same protocol as for the wild type.

To construct mutated dimers, the Haddock 2.4 tool [28,29] was used. PDB files were manually edited to rename zinc and copper atoms (Zn → Zn^2+^, Cu → Cu^2+^) and cleaned with the “pdb_selaltloc” tool from pdbtools [30] to remove alternate occupancies. The updated monomeric structures, now compatible with Haddock, were used to generate the following dimers: wt+wt, wt+A4V, wt+G41D, wt+G41S, wt+D76V, wt+G85R, wt+G93A, and wt+I104F.

In phase 2, molecular dynamics simulations using Amber were conducted to study conformational and property differences between the proteins. The dimers were obtained via Haddock [31,32] and refined using QuickPrep in MOE to correct metal charge errors. A Python script modified PDB files by changing the atom type (ME^2^→ ME) and residue name (ME^2^→ ME) to ensure proper Zn and Cu recognition by tleap.

The PDB files were cleaned to remove unrecognized hydrogen atoms, which were re-added in tleap following a protocol that included loading ff19SB, TIP3P water, and bivalent ion parameters; calculating system charge; solvating in a truncated octahedron box; neutralizing with Na^+^/Cl^−^ (determined via a MATLAB script which automatized a formula developed by Machado et al.); and saving topology and coordinates.

The 12-6-4 LJ-type nonbonded model for metal ions was implemented in Amber, adding the C4 term to the topology via ParmEd. Simulations began with two minimization steps, with and without restraints, to resolve steric clashes and prevent atomic overlaps.

In the first minimization phase, various parameters were employed to optimize the system’s energy. The parameter imin = 1 was used to activate the energy minimization process. The minimization algorithm followed a combined strategy of steepest descent and conjugate gradient, as specified by ntmin = 1. Initially, the steepest descent method was used to rapidly reduce the system’s energy, followed by the conjugate gradient method to refine the process more precisely and stably. The number of steepest descent cycles was set to ncyc = 10,000, while the maximum number of total minimization cycles (including those using the conjugate gradient method) was set to maxcyc = 20,000, ensuring proper energy convergence. To control molecular interactions during the simulation, ntp = 0 was specified, disabling pressure control to maintain a constant volume during minimization. Periodic boundary conditions were applied with ntb = 1, meaning the system was simulated within a cubic periodic box. A cutoff distance of 12.0 Å was defined with cut = 12.0, restricting nonbonded interactions to a maximum distance of 12 Å. This choice balances computational efficiency and accuracy for long-range interactions. Additionally, during this stage, restraints were applied to constrain specific parts of the structure. The parameter ntr = 1 activated the restraints, with a weight of restraint_wt = 10.0, determining the intensity of the constraints. The restraint mask restraintmask = ‘*&!:WAT&!@Na+&!:@Cl-&!@H’ excluded water molecules (WAT), sodium ions (Na^+^), chloride ions (Cl^−^), and hydrogen atoms from the restraints, applying them only to selected regions of the protein. Finally, the Ewald summation method was used to handle long-range electrostatic interactions, with nfft3 = 200 specifying the grid size for electrostatic force calculations.

In the second minimization phase, the parameters remained largely similar, with the key difference being the absence of restraints. Here, imin = 1 was again used to activate minimization, and the combined steepest descent and conjugate gradient approach was maintained with ntmin = 1. The number of steepest descent cycles remained at ncyc = 10,000, and the total minimization cycles were again maxcyc = 20,000, ensuring consistency with the first stage. Pressure control was still disabled with ntp = 0, and periodic boundary conditions were applied using ntb = 1 to simulate the system in a periodic box. The cutoff distance for nonbonded interactions remained 12.0 Å (cut = 12.0), and electrostatic interactions were again handled via the Ewald summation method with nfft3 = 200. The crucial difference in this phase was that ntr = 0, meaning no restraints were applied. This allowed the structure to move freely without restrictions, optimizing the system’s energy in a more flexible manner. The removal of restraints enabled the protein to adopt a more natural conformation, reducing artificial constraints that were previously imposed.

The second step involved heating, equilibration, and production simulation phases. These steps were executed using GPU-based commands.

During the heating phase, the simulation is configured for 500,000 integration steps, corresponding to a time of 1 ns, with a time step of 2 femtoseconds (fs). The seed for velocity generation is set to a random value, meaning a new seed is generated for each simulation run. Temperature control is managed by the Langevin thermostat, with a friction coefficient of 1.0 ps^−1^. Initially, the temperature is set to 0 K, but over the first 50,000 steps, it is gradually increased to 298 K. Coordinates are written to the trajectory file every 500 steps, and output information (such as potential energy, heat, etc.) is saved every 1000 steps. Nonbonded interactions between molecules are limited to a cutoff distance of 10 Å, and SHAKE constraints are applied to bonds involving hydrogen atoms to maintain molecular geometry fixed during the simulation. Additionally, positional restraints are applied to specific residues, such as water, sodium, chloride, and hydrogen, with a restraint force of 10.0. This approach ensures that during the heating phase, the protein structure does not undergo excessive modifications while being brought to the target temperature. Finally, the Ewald summation method is used for electrostatic interactions, with nfft3 = 200, which defines the FFT grid size for the Ewald sums.

During the equilibration phase, the simulation extends for another 500,000 steps (1 ns), maintaining the time step at 2 fs. During this phase, temperature control is handled by the Langevin thermostat, stabilizing the system at 298 K with a friction coefficient of 1.0 ps^−1^. Pressure control is activated using a Monte Carlo barostat, set to a reference pressure of 1 atm with a relaxation time of 2 ps, allowing the system to adjust to the target pressure conditions. Periodic boundary conditions are applied (NTB = 2), ensuring the simulation space is treated as continuous in three dimensions without edge limitations. Coordinates are written to the trajectory file every 500 steps, and output data are saved every 1000 steps. The cutoff for nonbonded interactions remains at 10 Å, and SHAKE constraints on hydrogen bonds are maintained. The simulation resumes from a restart file, ensuring continuity from the previous stage by reading velocities and coordinates from the restart file. Restraints are applied to selected residues with a restraint force of 5.0 to prevent the deformation of groups such as water, sodium, chloride, and hydrogen. The Ewald method is also employed for electrostatic interactions, with an FFT grid size of 200 points. This process allows the protein to reach an equilibrium state, where temperature and pressure are stabilized, and the structure is properly conditioned for the subsequent production phase.

In the production phase, the simulation is extended for 600 million integration steps, corresponding to a total time of 1.2 microseconds, with a time step of 2 femtoseconds (fs). The seed for velocity generation is set to a random value (ig = −1), meaning a new seed is generated for each simulation. Temperature control is managed by the Berendsen thermostat, with an initial temperature set to 298 K, which is gradually increased to 310 K over the first 1 million steps of the simulation. The thermostat relaxation time is set to 0.2 ps. Pressure control is activated (ntp = 1) and regulated using the Monte Carlo barostat, with a reference pressure of 1 atm and a relaxation time of 2 ps. Periodic boundary conditions are applied (ntb = 2), ensuring the continuous handling of the three-dimensional space without edge limitations. Nonbonded interactions between molecules are limited to a distance of 10 Å, while SHAKE constraints are applied to hydrogen-involving bonds to maintain their fixed geometry. The simulation resumes from a restart file (irest = 1), which contains initial coordinates and velocities (ntx = 5), ensuring continuity from previous simulations. Coordinate wrapping within the periodic box is disabled (iwrap = 0), preserving the molecules’ original spatial positions. Coordinates are written to the trajectory file every 50,000 steps, while output information, such as potential energy and temperature, is saved every 5000 steps. A restart file is written every 50,000 steps, allowing the simulation to be resumed in the case of interruption. The Ewald method is used for the calculation of long-range electrostatic interactions, with a 200-point FFT grid to improve the accuracy of Ewald summations.

At the end of the simulations, various analyses were performed using the CPPTRAJ tool in the Amber software package. First, the time evolution of RMSD and the radius of gyration over time, as well as the RMSF for each protein residue, were calculated. Subsequently, both the SASA for each residue and for each frame of the protein of interest, as well as the total SASA of the protein per frame were calculated (which is the one shown in the graphs). Finally, a conformational clustering was performed on the trajectory of each dimer, using the k-means algorithm to obtain 10 clusters.

Phase 3 focused on identifying the drugs that mitigate SOD1 misfolding and aggregation. Key candidates included PRG-A01 (Amisodin), which inhibits neuronal death and SOD1-TDP-43 aggregation in ALS murine models [33]; Telbivudine, a blood–brain barrier-permeable thymidine analog with potent anti-aggregation effects that reduces SOD1 toxicity [34]; and S-XL6, a stabilizer that binds to cysteine 111 to reinforce SOD1 dimers [35].

Ligand expansion via DrugBank’s Chemical Structure Search (threshold 0.6) identified additional candidates: Rotenone, Epigallocatechin gallate, and 30 Telbivudine analogs [36]. Additional selected compounds, including Baicalein, Lovastatin, Quercetin, and Simvastatin, are further discussed in the conclusions. These 39 ligands were screened for Lipinski’s Rule of Five compliance (Table 8) and docked using AutoDock VINA [37,38]. Blinded docking covered the entire protein due to unknown binding sites, except for S-XL6.

Docking involved the preparation of the PDBQT files, adjustment of metal ion charges, and definition of the grid box dimensions. To ensure consistency, the simulations were repeated, generating 100 poses per ligand (10 × 10, where the first ten refers to the number of docking iterations with Vina, and the second to the number of models produced per run). Finally, poses were clustered using DBSCAN in MATLAB based on an RMSD matrix computed in Python.

## 5. Conclusions

This study identified potential inhibitors targeting mutant SOD1, a key factor in ALS pathogenesis. Using AlphaFold2, molecular dynamics simulations (1200 ns), and blind docking, we analyzed seven mutated dimers and one wild-type dimer, revealing conformational differences. Notably, the wt+A4V dimer exhibited higher RMSD fluctuations, confirming previous findings [39].

The docking results identified PRG-A01, Baicalein, and Quercetin as the top ligands with the highest binding affinity, consistently interacting with specific regions of the protein—mostly on individual monomers. PRG-A01 and Quercetin also showed binding across the dimer interface, suggesting a possible stabilizing mechanism. S-XL6, Uridine, and Lovastatin which formed larger clusters were used to assess more information on the binding sites. These findings provide promising candidates for further evaluation.

### 5.1. Best Affinity Ligand Description

PRG-A01, also called amisodin, is an experimental inhibitor designed to prevent the misfolding and aggregation of Cu, Zn superoxide dismutase (SOD1). This mechanism is particularly relevant in pathological conditions where SOD1 misfolding contributes to disease progression. Currently, PRG-A01 is classified as an investigational drug. Baicalein is a flavonoid under investigation for its potential therapeutic effects. It has been studied in a Phase IIa clinical trial to evaluate the efficacy and safety of baicalein tablets in treating influenza-related fever in healthy adults. Quercetin is a flavonoid known for its ability to inhibit ribosyldihydronicotinamide dehydrogenase [quinone], an enzyme involved in the metabolism of toxic compounds. This inhibition may induce oxidative stress in Plasmodium species, the parasites responsible for malaria, potentially leading to their eradication [36]. The structural formula of those ligands can be seen in Table 9.

### 5.2. Larger Cluster-Forming Ligand Description

Larger cluster-forming ligands (Uridine, Lovastatin, and S-XL6) were used to assess more precise information about the binding sites around the SOD1 dimer.

Uridine is a ribonucleoside used, in combination with cytidine, to manage neuropsychiatric deficits associated with cerebrovascular diseases. Currently, uridine is approved for use in combination with cytidine for this therapeutic purpose. Lovastatin is an HMG-CoA reductase inhibitor approved for lowering low-density lipoprotein (LDL) cholesterol levels. It is used to reduce the risk of cardiovascular events, including myocardial infarction and stroke [36]. No information regarding S-XL6 is available in the DrugBank database; however, S-XL6 is a crosslinker developed to stabilize the dimer of superoxide dismutase 1 (SOD1), a protein involved in defense against oxidative stress. This ligand is designed to bind at the SOD1 dimer interface to prevent its dissociation, a phenomenon associated with the formation of toxic monomeric species implicated in neurodegenerative diseases such as amyotrophic lateral sclerosis (ALS) [35]. The structural formula of those ligands can be seen in Table 10.

For future studies, it would be highly beneficial, depending on the available computational resources, to create a much larger pool of compounds and analyze a greater number of mutations, considering that over 200 mutations impact the SOD1 gene and protein. Furthermore, for the docking procedure, it would be worthwhile to increase the number of poses to obtain more consistent data regarding each ligand’s binding region with the proteins, thereby identifying the binding sites. This approach could also involve applying molecular dynamics simulations to the best affinity dimer–ligand complexes to evaluate whether the presence of the ligand might have a beneficial effect, potentially influencing the protein’s conformational variability and/or stability.

## Figures and Tables

**Table 1:** Monomer amino acid sequences used for the wt and mutant variants of SOD1: mutated residues are highlighted in yellow.

Monomer	Sequence
wt	ATKAVCVLKGDGPVQGIINFEQKESNGPVKVWGSIKGLTEGLHGFHVHEFGDNTAGCTSAGPHFNPLSRKHGGPKDEERHVGDLGNVTADKDGVADVSIEDSVISLSGDHCIIGRTLVVHEKADDLGKGGNEESTKTGNAGSRLACGVIGIAQ
A4V	ATKVVCVLKGDGPVQGIINFEQKESNGPVKVWGSIKGLTEGLHGFHVHEFGDNTAGCTSAGPHFNPLSRKHGGPKDEERHVGDLGNVTADKDGVADVSIEDSVISLSGDHCIIGRTLVVHEKADDLGKGGNEESTKTGNAGSRLACGVIGIAQ
G41D	ATKAVCVLKGDGPVQGIINFEQKESNGPVKVWGSIKGLTEDLHGFHVHEFGDNTAGCTSAGPHFNPLSRKHGGPKDEERHVGDLGNVTADKDGVADVSIEDSVISLSGDHCIIGRTLVVHEKADDLGKGGNEESTKTGNAGSRLACGVIGIAQ
G41S	ATKAVCVLKGDGPVQGIINFEQKESNGPVKVWGSIKGLTESLHGFHVHEFGDNTAGCTSAGPHFNPLSRKHGGPKDEERHVGDLGNVTADKDGVADVSIEDSVISLSGDHCIIGRTLVVHEKADDLGKGGNEESTKTGNAGSRLACGVIGIAQ
D76V	ATKAVCVLKGDGPVQGIINFEQKESNGPVKVWGSIKGLTEGLHGFHVHEFGDNTAGCTSAGPHFNPLSRKHGGPKVEERHVGDLGNVTADKDGVADVSIEDSVISLSGDHCIIGRTLVVHEKADDLGKGGNEESTKTGNAGSRLACGVIGIAQ
G85R	ATKAVCVLKGDGPVQGIINFEQKESNGPVKVWGSIKGLTEGLHGFHVHEFGDNTAGCTSAGPHFNPLSRKHGGPKDEERHVGDLRNVTADKDGVADVSIEDSVISLSGDHCIIGRTLVVHEKADDLGKGGNEESTKTGNAGSRLACGVIGIAQ
G93A	ATKAVCVLKGDGPVQGIINFEQKESNGPVKVWGSIKGLTEGLHGFHVHEFGDNTAGCTSAGPHFNPLSRKHGGPKDEERHVGDLGNVTADKDAVADVSIEDSVISLSGDHCIIGRTLVVHEKADDLGKGGNEESTKTGNAGSRLACGVIGIAQ
I104F	ATKAVCVLKGDGPVQGIINFEQKESNGPVKVWGSIKGLTEGLHGFHVHEFGDNTAGCTSAGPHFNPLSRKHGGPKDEERHVGDLGNVTADKDGVADVSIEDSVFSLSGDHCIIGRTLVVHEKADDLGKGGNEESTKTGNAGSRLACGVIGIAQ

**Table 2:** Best affinity-ranked ligand 22 poses interacting with the wt+wt dimer. Note that the first model for each iteration has been selected.

Ligand 22		
Iteration 1 model 1	Iteration 2 model 1	Iteration 3 model 1
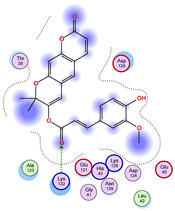	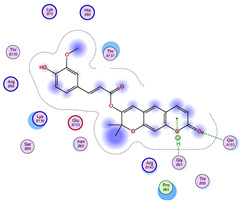	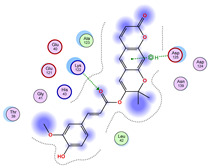
Iteration 4 model 1	Iteration 5 model 1	Iteration 6 model 1
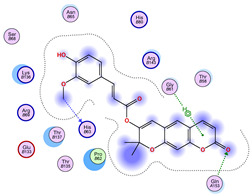	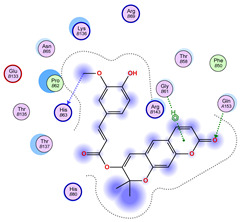	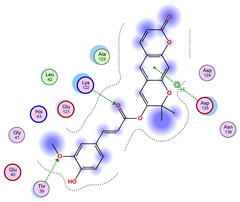
Iteration 7 model 1	Iteration 8 model 1	Iteration 9 model 1
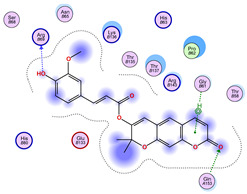	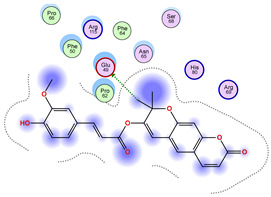	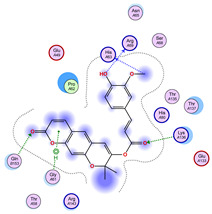
Iteration 10 model 1		
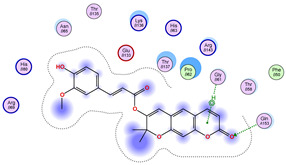		

**Table 3:** Best affinity-ranked ligand 36 poses interacting with the wt+wt dimer. Note that the first model for each iteration has been selected.

Ligand 36		
Iteration 1 model 1	Iteration 2 model 1	Iteration 3 model 1
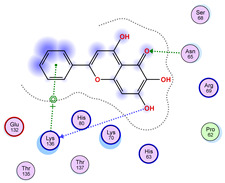	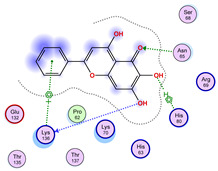	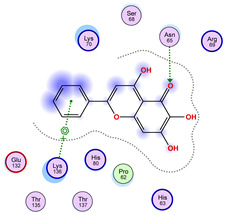
Iteration 4 model 1	Iteration 5 model 1	Iteration 6 model 1
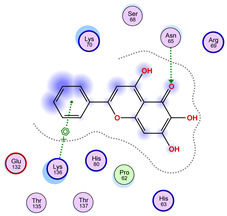	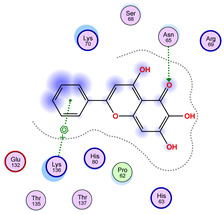	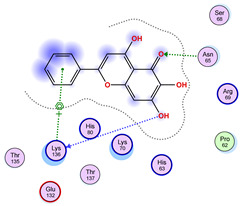
Iteration 7 model 1	Iteration 8 model 1	Iteration 9 model 1
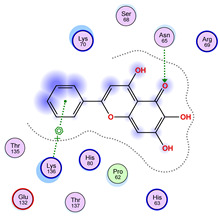	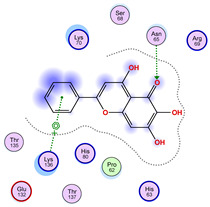	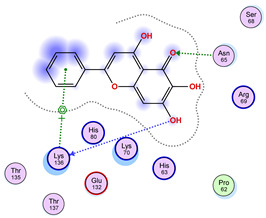
Iteration 10 model 1		
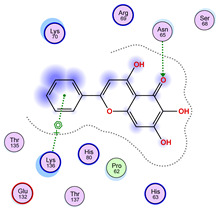		

**Table 4:** Best affinity-ranked ligand 38 poses interacting with the wt+wt dimer. Note that the first model for each iteration has been selected.

Ligand 38		
Iteration 1 model 1	Iteration 2 model 1	Iteration 3 model 1
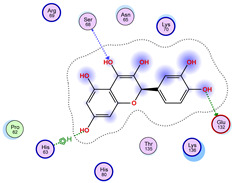	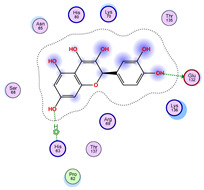	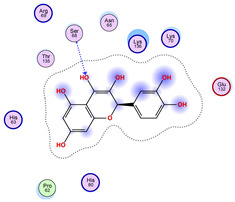
Iteration 4 model 1	Iteration 5 model 1	Iteration 6 model 1
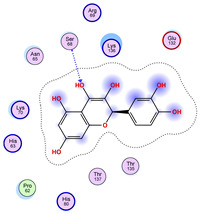	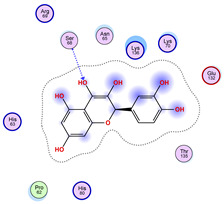	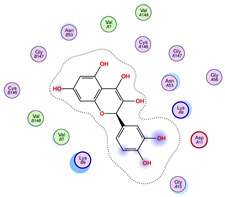
Iteration 7 model 1	Iteration 8 model 1	Iteration 9 model 1
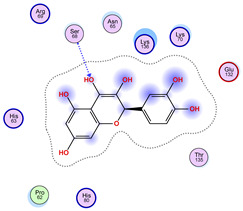	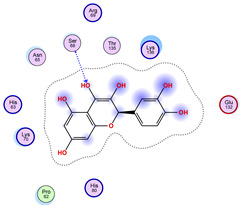	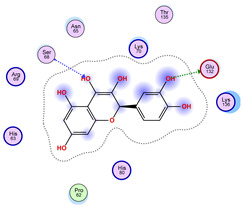
Iteration 10 model 1		
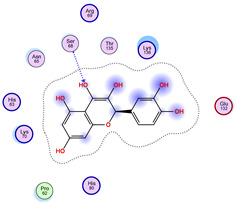		

**Table 5:** Ligand 22 interactions with wt+wt dimer: the models interacting with both monomers (chain A and B) are highlighted in blue.

Model	Atom Ligand (ID)	Atom Receptor	Res. Receptor	Chain (Monomer)	Interaction Type	Distance (Å)	E (kcal/mol)
Iteration 1							
1	O27 (20)	NZ	LYS 122	B	H-acceptor	3.08	−1.8
2	C31 (17)	OE2	GLU 49	B	H-donor	3.59	−0.6
3	—	—	—	—	—	—	—
4	O27 (20)	NZ	LYS 122	A	H-acceptor	3.38	−0.9
5	—	—	—	—	—	—	—
6	O15 (18)	NE	ARG 143	B	H-acceptor	3.16	−0.6
	6-ring	CA	GLY 141	B	pi-H	3.94	−0.5
7	C25 (30)	OG1	THR 137	B	H-donor	3.30	−0.6
	O27 (20)	CA	PRO 62	B	H-acceptor	3.37	−1.1
8	C25 (30)	SG	CYS 111	B	H-donor	3.71	−0.7
9	C25 (30)	OD1	ASN 139	B	H-donor	3.55	−0.7
	O6 (6)	ND2	ASN 65	B	H-acceptor	2.95	−1.6
10	O6 (6)	NE2	GLN 153	A	H-acceptor	3.21	−0.7
**Iteration 2**							
**Model**	**Atom Ligand (ID)**	**Atom Receptor**	**Res. Receptor**	**Chain (monomer)**	**Interaction type**	**Distance (Å)**	**E (kcal/mol)**
1	O6 (6)	NE2	GLN 153	A	H-acceptor	3.13	−1.3
	6-ring	CA	GLY 61	B	pi-H	3.61	−2.4
2	6-ring	CA	GLY 61	A	pi-H	3.36	−0.8
3	O27 (20)	NZ	LYS 122	A	H-acceptor	3.18	−1.8
4	—	—	—	—	—	—	—
5	C31 (17)	OE2	GLU 49	B	H-donor	3.28	−0.7
6	—	—	—	—	—	—	—
7	O6 (6)	NE1	TRP 32	B	H-acceptor	3.23	−0.6
8	O26 (31)	OD2	ASP 11	B	H-donor	3.14	−0.8
	6-ring	NZ	LYS 36	A	pi-cation	4.22	−0.9
9	C3 (3)	OE2	GLU 49	B	H-donor	3.49	−1.5
	C30 (16)	O	ARG 69	B	H-donor	3.68	−0.5
	O27 (20)	N	HIS 63	B	H-acceptor	3.24	−1.2
10	C3 (3)	OE2	GLU 49	B	H-donor	3.69	−1.1
	O27 (20)	CE1	HIS 80	B	H-acceptor	3.66	−0.5
**Iteration 3**							
**Model**	**Atom Ligand (ID)**	**Atom Receptor**	**Res. Receptor**	**Chain (monomer)**	**Interaction type**	**Distance (Å)**	**E (kcal/mol)**
1	O27 (20)	NZ	LYS 122	A	H-acceptor	3.17	−1.5
	6-ring	CA	ASP 125	A	pi-H	3.64	−0.6
2	O26 (31)	O	GLU 40	B	H-donor	2.89	−1.4
	O27 (20)	NZ	LYS 122	B	H-acceptor	3.19	−1.1
3	O27 (20)	CA	PRO 66	A	H-acceptor	3.57	−0.5
	O24 (29)	NH2	ARG 115	A	H-acceptor	3.10	−1.4
4	—	—	—	—	—	—	—
5	C25 (30)	O	ASP 109	B	H-donor	3.77	−0.5
	O27 (20)	CA	PRO 66	B	H-acceptor	3.64	−0.5
6	O27 (20)	CA	PRO 62	A	H-acceptor	3.35	−1.1
	6-ring	CG	LYS 136	A	pi-H	3.64	−0.5
7	C7 (7)	OG1	THR 137	B	H-donor	3.52	−0.6
	6-ring	CA	GLY 141	B	pi-H	3.78	−0.5
8	O6 (6)	NE1	TRP 32	B	H-acceptor	3.07	−1.8
9	—	—	—	—	—	—	—
10	O24 (29)	ND2	ASN 65	A	H-acceptor	3.02	−1.5
**Iteration 4**							
**Model**	**Atom Ligand (ID)**	**Atom Receptor**	**Res. Receptor**	**Chain (monomer)**	**Interaction type**	**Distance (Å)**	**E (kcal/mol)**
1	C25 (30)	O	HIS 63	B	H-donor	3.70	−0.5
	O6 (6)	NE2	GLN 153	A	H-acceptor	3.24	−0.8
	6-ring	CA	GLY 61	B	pi-H	3.66	−1.8
2	C25 (30)	O	HIS 63	A	H-donor	3.65	−0.7
	O6 (6)	NE2	GLN 153	B	H-acceptor	3.23	−1.6
3	C8 (8)	O	ILE 17	B	H-donor	3.32	−0.5
	C12 (12)	O	SER 34	B	H-donor	3.58	−0.5
4	C25 (30)	O	ASP 92	A	H-donor	3.48	−0.8
5	C31 (17)	OE2	GLU 49	B	H-donor	3.51	−0.5
6	C25 (30)	OG1	THR 137	A	H-donor	3.45	−0.6
	O27 (20)	CA	PRO 62	A	H-acceptor	3.51	−0.8
	6-ring	CG	LYS 136	A	pi-H	3.68	−0.5
7	O6 (6)	CA	GLY 33	B	H-acceptor	3.65	−0.7
	O27 (20)	NZ	LYS 36	B	H-acceptor	2.98	−2.7
8	C3 (3)	O	HIS 63	B	H-donor	3.55	−0.7
	C8 (8)	O	HIS 63	B	H-donor	3.66	−0.5
	C25 (30)	O	ASP 109	B	H-donor	3.53	−0.7
	O27 (20)	CA	PRO 66	B	H-acceptor	3.50	−0.6
9	C25 (30)	OE1	GLN 153	A	H-donor	3.33	−0.5
	6-ring	CA	GLY 61	B	pi-H	3.80	−1.0
10	O27 (20)	CA	PRO 62	B	H-acceptor	3.61	−0.7
**Iteration 5**							
**Model**	**Atom Ligand (ID)**	**Atom Receptor**	**Res. Receptor**	**Chain (monomer)**	**Interaction type**	**Distance (Å)**	**E (kcal/mol)**
1	C25 (30)	O	HIS 63	B	H-donor	3.71	−0.6
	O6 (6)	NE2	GLN 153	A	H-acceptor	3.11	−1.6
	6-ring	CA	GLY 61	B	pi-H	3.67	−0.6
2	O27 (20)	NZ	LYS 122	B	H-acceptor	3.21	−1.2
	6-ring	CA	ASP 125	B	pi-H	3.68	−0.7
3	O27 (20)	NZ	LYS 122	A	H-acceptor	3.07	−2.0
4	O6 (6)	NE2	GLN 153	B	H-acceptor	3.08	−1.9
	O27 (20)	CD	LYS 136	A	H-acceptor	3.58	−0.5
5	C31 (17)	OE1	GLU 49	A	H-donor	3.55	−0.5
6	—	—	—	—	—	—	—
7	C8 (8)	O	HIS 63	B	H-donor	3.51	−0.5
	O27 (20)	CA	PRO 66	B	H-acceptor	3.57	−0.5
8	—	—	—	—	—	—	—
9	C8 (8)	O	ILE 17	B	H-donor	3.12	−0.6
	C12 (12)	O	SER 34	B	H-donor	3.46	−0.6
10	O6 (6)	ND2	ASN 53	B	H-acceptor	3.12	−2.4
**Iteration 6**							
**Model**	**Atom Ligand (ID)**	**Atom Receptor**	**Res. Receptor**	**Chain (monomer)**	**Interaction type**	**Distance (Å)**	**E (kcal/mol)**
1	O27 (20)	NZ	LYS 122	A	H-acceptor	3.17	−1.5
	O24 (29)	OG1	THR 39	A	H-acceptor	2.84	−1.1
	6-ring	CA	ASP 125	A	pi-H	3.68	−0.7
2	C25 (30)	O	HIS 63	A	H-donor	3.82	−0.6
	6-ring	CA	GLY 61	A	pi-H	3.56	−1.1
3	O27 (20)	NZ	LYS 122	B	H-acceptor	3.12	−1.6
	O24 (29)	OG1	THR 39	B	H-acceptor	2.87	−0.9
4	—	—	—	—	—	—	—
5	—	—	—	—	—	—	—
6	O6 (6)	NE2	GLN 153	A	H-acceptor	2.99	−3.0
7	—	—	—	—	—	—	—
8	O27 (20)	CA	PRO 66	B	H-acceptor	3.47	−0.7
9	C3 (3)	O	HIS 63	A	H-donor	3.46	−0.6
10	C25 (30)	OD1	ASN 139	B	H-donor	3.70	−0.5
	O6 (6)	ND2	ASN 65	B	H-acceptor	2.93	−2.1
**Iteration 7**							
**Model**	**Atom Ligand (ID)**	**Atom Receptor**	**Res. Receptor**	**Chain (monomer)**	**Interaction type**	**Distance (Å)**	**E (kcal/mol)**
1	O26 (31)	O	ARG 69	B	H-donor	2.88	−0.7
	O6 (6)	NE2	GLN 153	A	H-acceptor	3.27	−0.6
	6-ring	CA	GLY 61	B	pi-H	3.57	−1.6
2	C25 (30)	O	HIS 63	A	H-donor	3.59	−0.7
	6-ring	CA	GLY 61	A	pi-H	3.38	−1.0
3	O27 (20)	NZ	LYS 122	A	H-acceptor	3.20	−1.7
4	C25 (30)	O	ASP 92	A	H-donor	3.83	−0.5
5	—	—	—	—	—	—	—
6	6-ring	6-ring	TRP 32	A	pi-pi	3.69	−0.0
	6-ring	5-ring	TRP 32	A	pi-pi	3.62	−0.0
7	C7 (7)	OG1	THR 137	B	H-donor	3.48	−0.6
	C25 (30)	O	ARG 143	B	H-donor	3.43	−0.5
	6-ring	CA	GLY 141	B	pi-H	3.70	−0.5
	6-ring	N	ARG 143	B	pi-H	4.20	−0.6
8	C25 (30)	O	HIS 63	A	H-donor	3.67	−0.7
9	—	—	—	—	—	—	—
10	—	—	—	—	—	—	—
**Iteration 8**							
**Model**	**Atom Ligand (ID)**	**Atom Receptor**	**Res. Receptor**	**Chain (monomer)**	**Interaction type**	**Distance (Å)**	**E (kcal/mol)**
1	C31 (17)	OE1	GLU 49	A	H-donor	3.47	−0.5
2	O27 (20)	CE	LYS 122	A	H-acceptor	3.46	−1.3
3	O27 (20)	NZ	LYS 122	B	H-acceptor	3.23	−1.0
4	C25 (30)	O	ASP 92	A	H-donor	3.71	−0.6
5	6-ring	6-ring	TRP 32	A	pi-pi	3.70	−0.0
	6-ring	5-ring	TRP 32	A	pi-pi	3.62	−0.0
6	O27 (20)	CA	PRO 62	B	H-acceptor	3.44	−0.9
	6-ring	CG	LYS 136	B	pi-H	3.61	−0.5
7	—	—	—	—	—	—	—
8	O15 (18)	NH2	ARG 143	B	H-acceptor	2.94	−1.3
9	O6 (6)	NZ	LYS 9	B	H-acceptor	3.25	−2.1
10	—	—	—	—	—	—	—
**Iteration 9**							
**Model**	**Atom Ligand (ID)**	**Atom Receptor**	**Res. Receptor**	**Chain (monomer)**	**Interaction type**	**Distance (Å)**	**E (kcal/mol)**
1	C25 (30)	O	HIS 63	A	H-donor	3.57	−0.7
	O26 (31)	O	ARG 69	A	H-donor	2.73	−1.3
	O6 (6)	NE2	GLN 153	B	H-acceptor	3.22	−1.4
	O27 (20)	CD	LYS 136	A	H-acceptor	3.56	−0.5
	6-ring	CA	GLY 61	A	pi-H	3.82	−0.6
2	C25 (30)	O	HIS 63	B	H-donor	3.64	−0.7
	O6 (6)	NE2	GLN 153	A	H-acceptor	2.82	−3.8
3	O27 (20)	CE	LYS 122	A	H-acceptor	3.47	−1.2
4	C8 (8)	O	ILE 17	B	H-donor	3.18	−0.6
	C12 (12)	O	SER 34	B	H-donor	3.49	−0.6
5	—	—	—	—	—	—	—
6	C25 (30)	O	ASP 92	A	H-donor	3.69	−0.5
7	O26 (31)	O	ASN 53	A	H-donor	2.85	−3.7
	O27 (20)	NZ	LYS 36	B	H-acceptor	2.95	−2.8
8	C25 (30)	O	HIS 63	A	H-donor	3.25	−0.9
	O15 (18)	NH2	ARG 143	A	H-acceptor	2.86	−1.3
9	—	—	—	—	—	—	—
10	C31 (17)	OE1	GLU 49	A	H-donor	3.54	−0.5
**Iteration 10**							
**Model**	**Atom Ligand (ID)**	**Atom Receptor**	**Res. Receptor**	**Chain (monomer)**	**Interaction type**	**Distance (Å)**	**E (kcal/mol)**
1	O6 (6)	NE2	GLN 153	A	H-acceptor	3.14	−1.2
	6-ring	CA	GLY 61	B	pi-H	3.58	−2.1
2	O6 (6)	NE2	GLN 153	A	H-acceptor	3.24	−0.5
	6-ring	CA	GLY 61	B	pi-H	3.56	−1.0
3	—	—	—	—	—	—	—
4	C31 (17)	OE2	GLU 49	B	H-donor	3.37	−0.7
5	O26 (31)	O	GLU 40	A	H-donor	2.93	−1.3
	O27 (20)	NZ	LYS 122	A	H-acceptor	3.19	−1.5
6	C8 (8)	O	ILE 17	B	H-donor	3.18	−0.6
	C12 (12)	O	SER 34	B	H-donor	3.48	−0.6
7	C25 (30)	OG1	THR 137	B	H-donor	3.50	−0.6
	O27 (20)	CA	PRO 62	B	H-acceptor	3.64	−0.7
	6-ring	CG	LYS 136	B	pi-H	3.65	−0.5
8	—	—	—	—	—	—	—
9	O6 (6)	NZ	LYS 9	A	H-acceptor	3.21	−1.8
	6-ring	CG2	ILE 17	A	pi-H	3.55	−0.6
10	C25 (30)	O	ASP 92	A	H-donor	3.68	−0.5

**Table 6:** Ligand 36 interactions with wt+wt dimer: the models interacting with both monomers (chain A and B) are highlighted in blue.

Iteration 1							
Model	Atom Ligand (ID)	Atom Receptor	Res. Receptor	Chain (Monomer)	Interaction Type	Distance (Å)	E (kcal/mol)
1	O14 (19)	O	LYS 136	B	H-donor	3.22	−0.6
	O10 (10)	CB	ASN 65	B	H-acceptor	3.26	−0.6
	O10 (10)	ND2	ASN 65	B	H-acceptor	3.05	−1.4
	6-ring	NZ	LYS 136	B	pi-cation	4.74	−2.2
2	C16 (14)	O	GLN 15	A	H-donor	3.45	−0.5
3	O10 (10)	CB	ASN 65	A	H-acceptor	3.26	−0.6
	O10 (10)	ND2	ASN 65	A	H-acceptor	3.01	−1.7
	O15 (21)	5-ring	HIS 80	A	H-pi	4.35	−1.3
	6-ring	NZ	LYS 136	A	pi-cation	4.90	−2.0
4	O10 (10)	CB	PRO 62	A	H-acceptor	3.06	−0.5
	O10 (10)	N	HIS 63	A	H-acceptor	3.07	−0.6
	6-ring	ND2	ASN 65	A	pi-H	3.64	−0.5
5	O10 (10)	CB	PRO 62	B	H-acceptor	3.11	−0.5
	O10 (10)	N	HIS 63	B	H-acceptor	3.10	−0.8
	6-ring	ND2	ASN 65	B	pi-H	3.58	−0.5
6	O10 (10)	ND2	ASN 65	B	H-acceptor	2.80	−5.1
	O12 (12)	N	HIS 63	B	H-acceptor	3.16	−0.8
7	6-ring	ND2	ASN 65	A	pi-H	3.59	−0.8
	6-ring	NZ	LYS 70	A	pi-cation	4.04	−1.4
8	6-ring	ND2	ASN 65	B	pi-H	3.62	−0.8
9	O10 (10)	NH1	ARG 115	B	H-acceptor	3.05	−1.8
	O10 (10)	NH2	ARG 115	B	H-acceptor	3.10	−3.7
10	C11 (11)	O	GLN 15	A	H-donor	3.34	−0.8
	O12 (12)	O	GLN 15	A	H-donor	3.70	−0.5
**Iteration 2**							
**Model**	**Atom Ligand (ID)**	**Atom Receptor**	**Res. Receptor**	**Chain (monomer)**	**Interaction type**	**Distance (Å)**	**E (kcal/mol)**
1	O14 (19)	O	LYS 136	B	H-donor	3.17	−0.6
	O10 (10)	ND2	ASN 65	B	H-acceptor	3.02	−1.5
	6-ring	NZ	LYS 136	B	pi-cation	4.88	−2.2
2	O10 (10)	CB	ASN 65	A	H-acceptor	3.25	−0.6
	O10 (10)	ND2	ASN 65	A	H-acceptor	3.00	−1.6
	6-ring	NZ	LYS 136	A	pi-cation	4.84	−2.1
	O14 (19)	5-ring	HIS 80	A	H-pi	4.24	−1.2
3	O10 (10)	ND2	ASN 65	B	H-acceptor	2.84	−5.1
	O14 (19)	5-ring	HIS 80	B	H-pi	4.39	−1.4
	6-ring	NZ	LYS 136	B	pi-cation	4.88	−2.0
4	O10 (10)	CB	ASN 65	A	H-acceptor	3.27	−0.6
	O10 (10)	ND2	ASN 65	A	H-acceptor	3.01	−1.5
	6-ring	NZ	LYS 136	A	pi-cation	4.93	−2.0
5	O10 (10)	CB	ASN 65	B	H-acceptor	3.27	−0.6
	O10 (10)	ND2	ASN 65	B	H-acceptor	3.06	−1.6
	6-ring	NZ	LYS 136	B	pi-cation	4.82	−2.2
6	O10 (10)	ND2	ASN 65	A	H-acceptor	2.86	−4.8
	O14 (19)	5-ring	HIS 80	A	H-pi	4.31	−1.3
7	O10 (10)	ND2	ASN 65	B	H-acceptor	2.93	−5.2
	O14 (19)	5-ring	HIS 80	B	H-pi	4.25	−1.4
8	O10 (10)	CB	ASN 65	A	H-acceptor	3.25	−0.6
	O10 (10)	ND2	ASN 65	A	H-acceptor	3.04	−1.5
9	O10 (10)	CB	ASN 65	B	H-acceptor	3.28	−0.6
	O10 (10)	ND2	ASN 65	B	H-acceptor	3.05	−1.5
10	O14 (19)	O	LYS 136	A	H-donor	3.34	−0.6
	6-ring	NZ	LYS 136	A	pi-cation	4.87	−2.2
**Iteration 3**							
**Model**	**Atom Ligand (ID)**	**Atom Receptor**	**Res. Receptor**	**Chain (monomer)**	**Interaction type**	**Distance (Å)**	**E (kcal/mol)**
1	O10 (10)	ND2	ASN 65	B	H-acceptor	3.09	−1.5
	6-ring	CA	GLY 141	B	pi-H	3.82	−0.5
	6-ring	CD	LYS 136	B	pi-H	3.80	−0.6
2	O10 (10)	ND2	ASN 65	A	H-acceptor	3.03	−1.5
	6-ring	CA	GLY 141	A	pi-H	3.77	−0.6
	6-ring	CD	LYS 136	A	pi-H	3.81	−0.6
3	O10 (10)	ND2	ASN 65	B	H-acceptor	3.04	−1.5
	6-ring	CA	GLY 141	B	pi-H	3.77	−0.6
	6-ring	CD	LYS 136	B	pi-H	3.79	−0.6
4	O10 (10)	ND2	ASN 65	A	H-acceptor	2.97	−1.5
	6-ring	CA	GLY 141	A	pi-H	3.76	−0.5
	6-ring	CD	LYS 136	A	pi-H	3.78	−0.5
5	O10 (10)	ND2	ASN 65	B	H-acceptor	2.91	−1.6
	6-ring	CA	GLY 141	B	pi-H	3.73	−0.6
	6-ring	CD	LYS 136	B	pi-H	3.79	−0.5
6	O10 (10)	ND2	ASN 65	A	H-acceptor	2.93	−1.5
	6-ring	CA	GLY 141	A	pi-H	3.71	−0.6
	6-ring	CD	LYS 136	A	pi-H	3.74	−0.5
7	O10 (10)	ND2	ASN 65	B	H-acceptor	2.92	−1.5
	6-ring	CA	GLY 141	B	pi-H	3.76	−0.6
	6-ring	CD	LYS 136	B	pi-H	3.75	−0.6
8	O10 (10)	ND2	ASN 65	A	H-acceptor	2.95	−1.4
	6-ring	CA	GLY 141	A	pi-H	3.78	−0.6
	6-ring	CD	LYS 136	A	pi-H	3.72	−0.6
9	O10 (10)	ND2	ASN 65	B	H-acceptor	2.90	−1.5
	6-ring	CA	GLY 141	B	pi-H	3.75	−0.6
	6-ring	CD	LYS 136	B	pi-H	3.70	−0.6
10	O10 (10)	ND2	ASN 65	A	H-acceptor	2.91	−1.5
	6-ring	CA	GLY 141	A	pi-H	3.74	−0.6
	6-ring	CD	LYS 136	A	pi-H	3.69	−0.6
**Iteration 4**							
**Model**	**Atom Ligand (ID)**	**Atom Receptor**	**Res. Receptor**	**Chain (monomer)**	**Interaction type**	**Distance (Å)**	**E (kcal/mol)**
1	O10 (10)	CB	ASN 65	B	H-acceptor	3.26	−0.6
	O10 (10)	ND2	ASN 65	B	H-acceptor	3.05	−1.4
	6-ring	NZ	LYS 136	B	pi-cation	4.73	−2.3
2	O10 (10)	CB	ASN 65	A	H-acceptor	3.27	−0.6
	O10 (10)	ND2	ASN 65	A	H-acceptor	3.03	−1.6
	6-ring	NZ	LYS 136	A	pi-cation	4.89	−2.0
3	C17 (18)	O	GLN 15	A	H-donor	3.50	−0.5
4	O14 (19)	O	ASN 65	A	H-donor	3.18	−1.0
	O10 (10)	CB	PRO 62	A	H-acceptor	3.05	−0.5
	O10 (10)	N	HIS 63	A	H-acceptor	3.07	−0.6
	6-ring	ND2	ASN 65	A	pi-H	3.64	−0.5
5	O15 (21)	O	HIS 63	B	H-donor	2.85	−0.7
	O10 (10)	CB	PRO 62	B	H-acceptor	3.12	−0.5
	O10 (10)	N	HIS 63	B	H-acceptor	3.11	−0.7
	6-ring	ND2	ASN 65	B	pi-H	3.58	−0.5
6	O10 (10)	ND2	ASN 65	A	H-acceptor	2.81	−5.1
	O12 (12)	N	HIS 63	A	H-acceptor	3.13	−0.8
7	O10 (10)	ND2	ASN 65	B	H-acceptor	2.80	−5.1
	O12 (12)	N	HIS 63	B	H-acceptor	3.15	−0.8
	O15 (21)	5-ring	HIS 80	B	H-pi	4.83	−2.0
8	O12 (12)	O	ASN 53	B	H-donor	2.96	−0.5
	6-ring	NZ	LYS 9	A	pi-cation	3.61	−4.0
9	—	—	—	—	—	—	—
10	—	—	—	—	—	—	—
**Iteration 5**							
**Model**	**Atom Ligand (ID)**	**Atom Receptor**	**Res. Receptor**	**Chain (monomer)**	**Interaction type**	**Distance (Å)**	**E (kcal/mol)**
1	O10 (10)	CB	ASN 65	A	H-acceptor	3.27	−0.6
	O10 (10)	ND2	ASN 65	A	H-acceptor	3.03	−1.4
	6-ring	NZ	LYS 136	A	pi-cation	4.70	−2.3
2	O10 (10)	CB	ASN 65	B	H-acceptor	3.27	−0.6
	O10 (10)	ND2	ASN 65	B	H-acceptor	3.04	−1.4
	6-ring	NZ	LYS 136	B	pi-cation	4.85	−2.1
3	O10 (10)	ND2	ASN 65	A	H-acceptor	2.84	−5.1
	O12 (12)	N	HIS 63	A	H-acceptor	3.16	−0.8
4	O10 (10)	ND2	ASN 65	B	H-acceptor	2.83	−5.1
	O12 (12)	N	HIS 63	B	H-acceptor	3.15	−0.8
5	O14 (19)	O	ASN 65	B	H-donor	3.14	−1.0
	O10 (10)	CB	PRO 62	B	H-acceptor	3.05	−0.5
	O10 (10)	N	HIS 63	B	H-acceptor	3.04	−0.6
	6-ring	ND2	ASN 65	B	pi-H	3.67	−0.5
6	O14 (19)	O	ASN 65	A	H-donor	3.18	−1.0
	O10 (10)	CB	PRO 62	A	H-acceptor	3.05	−0.5
	O10 (10)	N	HIS 63	A	H-acceptor	3.06	−0.6
	6-ring	ND2	ASN 65	A	pi-H	3.63	−0.5
7	O12 (12)	O	ASN 53	A	H-donor	2.99	−0.5
	6-ring	NZ	LYS 9	B	pi-cation	3.62	−3.9
8	C17 (18)	O	GLN 15	B	H-donor	3.46	−0.5
9	O15 (21)	O	HIS 63	B	H-donor	2.86	−0.7
	O10 (10)	CB	PRO 62	B	H-acceptor	3.12	−0.5
	O10 (10)	N	HIS 63	B	H-acceptor	3.12	−0.7
	6-ring	ND2	ASN 65	B	pi-H	3.59	−0.5
10	—	—	—	—	—	—	—
**Iteration 6**							
**Model**	**Atom Ligand (ID)**	**Atom Receptor**	**Res. Receptor**	**Chain (monomer)**	**Interaction type**	**Distance (Å)**	**E (kcal/mol)**
1	O12 (12)	O	ASN 53	A	H-donor	3.04	−0.5
	O10 (10)	ND2	ASN 65	A	H-acceptor	2.81	−5.3
	6-ring	ND2	ASN 65	A	pi-H	3.66	−0.5
2	O10 (10)	ND2	ASN 65	B	H-acceptor	2.82	−5.2
	O12 (12)	O	ASN 53	B	H-donor	3.04	−0.5
	6-ring	ND2	ASN 65	B	pi-H	3.63	−0.5
3	O10 (10)	CB	ASN 65	A	H-acceptor	3.26	−0.6
	O10 (10)	ND2	ASN 65	A	H-acceptor	3.03	−1.4
	6-ring	NZ	LYS 136	A	pi-cation	4.65	−2.2
4	O10 (10)	CB	ASN 65	B	H-acceptor	3.29	−0.6
	O10 (10)	ND2	ASN 65	B	H-acceptor	3.01	−1.4
	6-ring	NZ	LYS 136	B	pi-cation	4.72	−2.1
5	O10 (10)	N	HIS 63	B	H-acceptor	3.09	−0.6
	6-ring	ND2	ASN 65	B	pi-H	3.64	−0.5
	O10 (10)	CB	PRO 62	B	H-acceptor	3.10	−0.5
6	O10 (10)	N	HIS 63	A	H-acceptor	3.07	−0.6
	6-ring	ND2	ASN 65	A	pi-H	3.58	−0.5
	O10 (10)	CB	PRO 62	A	H-acceptor	3.11	−0.5
7	O12 (12)	O	ASN 53	B	H-donor	2.97	−0.5
	6-ring	NZ	LYS 9	A	pi-cation	3.70	−3.9
8	O10 (10)	CB	ASN 65	B	H-acceptor	3.22	−0.6
	O10 (10)	ND2	ASN 65	B	H-acceptor	2.95	−1.5
	6-ring	NZ	LYS 136	B	pi-cation	4.40	−2.2
9	C17 (18)	O	GLN 15	B	H-donor	3.30	−0.5
10	O10 (10)	ND2	ASN 65	A	H-acceptor	2.83	−5.3
	O12 (12)	O	ASN 53	A	H-donor	3.01	−0.5
	6-ring	ND2	ASN 65	A	pi-H	3.66	−0.5
**Iteration 7**							
**Model**	**Atom Ligand (ID)**	**Atom Receptor**	**Res. Receptor**	**Chain (monomer)**	**Interaction type**	**Distance (Å)**	**E (kcal/mol)**
1	O10	CB	ASN 65	B	H-acceptor	3.26	−0.6
	O10	ND2	ASN 65	B	H-acceptor	3.04	−1.4
	6-ring	NZ	LYS 136	B	pi-cation	4.72	−2.3
2	C16	O	GLN 15	A	H-donor	3.45	−0.5
	O14	O	THR 54	B	H-donor	3.04	−1.9
3	O10	CB	ASN 65	A	H-acceptor	3.27	−0.6
	O10	ND2	ASN 65	A	H-acceptor	3.04	−1.7
	6-ring	NZ	LYS 136	A	pi-cation	4.89	−2.1
4	O10	CB	PRO 62	B	H-acceptor	3.11	−0.5
	O10	N	HIS 63	B	H-acceptor	3.09	−0.8
	6-ring	ND2	ASN 65	B	pi-H	3.59	−0.5
5	O10	CB	PRO 62	A	H-acceptor	3.06	−0.5
	O10	N	HIS 63	A	H-acceptor	3.07	−0.6
	6-ring	ND2	ASN 65	A	pi-H	3.64	−0.5
6	O10	ND2	ASN 65	A	H-acceptor	2.80	−5.0
	O12	N	HIS 63	A	H-acceptor	3.17	−0.8
7	6-ring	NZ	LYS 9	A	pi-cation	3.63	−1.2
8	O14	O	THR 135	A	H-donor	3.15	−0.9
9	O10	NH1	ARG 115	B	H-acceptor	3.05	−1.8
	O10	NH2	ARG 115	B	H-acceptor	3.10	−3.7
10	O12	NZ	LYS 122	A	H-acceptor	3.22	−1.2
**Iteration 8**							
**Model**	**Atom Ligand (ID)**	**Atom Receptor**	**Res. Receptor**	**Chain (monomer)**	**Interaction type**	**Distance (Å)**	**E (kcal/mol)**
1	O12	NZ	LYS 122	A	H-acceptor	3.07	−1.7
	O12	CD	LYS 122	A	H-acceptor	3.59	−0.4
2	O10	CB	PRO 62	A	H-acceptor	3.06	−0.5
	O10	N	HIS 63	A	H-acceptor	3.09	−0.6
	6-ring	ND2	ASN 65	A	pi-H	3.64	−0.6
3	O14	N	HIS 63	B	H-acceptor	3.15	−0.9
	O14	CB	PRO 62	B	H-acceptor	3.27	−0.7
	6-ring	ND2	ASN 65	B	pi-H	3.59	−0.4
4	O10	NH1	ARG 115	A	H-acceptor	3.06	−1.4
	O10	NH2	ARG 115	A	H-acceptor	3.14	−2.8
5	6-ring	NZ	LYS 9	A	pi-cation	3.92	−1.1
6	O12	CD	LYS 122	A	H-acceptor	3.25	−0.6
7	O10	O	THR 54	A	H-donor	3.09	−0.9
8	O12	NE	ARG 115	A	H-acceptor	3.02	−1.6
9	O12	CD	LYS 122	B	H-acceptor	3.41	−0.5
10	O12	NZ	LYS 122	B	H-acceptor	3.28	−1.1
**Iteration 9**							
**Model**	**Atom Ligand (ID)**	**Atom Receptor**	**Res. Receptor**	**Chain (monomer)**	**Interaction type**	**Distance (Å)**	**E (kcal/mol)**
1	O12	NZ	LYS 122	A	H-acceptor	3.09	−1.5
	O12	CD	LYS 122	A	H-acceptor	3.63	−0.5
2	O10	CB	PRO 62	A	H-acceptor	3.08	−0.5
	O10	N	HIS 63	A	H-acceptor	3.10	−0.6
	6-ring	ND2	ASN 65	A	pi-H	3.60	−0.5
3	O14	N	HIS 63	B	H-acceptor	3.16	−1.0
	O14	CB	PRO 62	B	H-acceptor	3.25	−0.6
	6-ring	ND2	ASN 65	B	pi-H	3.62	−0.5
4	O10	NH1	ARG 115	A	H-acceptor	3.09	−1.3
	O10	NH2	ARG 115	A	H-acceptor	3.15	−2.9
5	6-ring	NZ	LYS 9	A	pi-cation	3.94	−1.2
6	O12	CD	LYS 122	A	H-acceptor	3.27	−0.7
7	O10	O	THR 54	A	H-donor	3.06	−1.0
8	O12	NE	ARG 115	A	H-acceptor	3.05	−1.7
9	O12	CD	LYS 122	B	H-acceptor	3.43	−0.6
10	O12	NZ	LYS 122	B	H-acceptor	3.31	−1.3
**Iteration 10**							
**Model**	**Atom Ligand (ID)**	**Atom Receptor**	**Res. Receptor**	**Chain (monomer)**	**Interaction type**	**Distance (Å)**	**E (kcal/mol)**
1	O10 (10)	CB	ASN 65	B	H-acceptor	3.26	−0.6
	O10 (10)	ND2	ASN 65	B	H-acceptor	3.04	−1.4
	6-ring	NZ	LYS 136	B	pi-cation	4.73	−2.3
2	C16 (14)	O	GLN 15	A	H-donor	3.45	−0.5
3	O10 (10)	CB	ASN 65	A	H-acceptor	3.26	−0.6
	O10 (10)	ND2	ASN 65	A	H-acceptor	3.04	−1.6
	6-ring	NZ	LYS 136	A	pi-cation	4.88	−2.1
4	O10 (10)	CB	PRO 62	A	H-acceptor	3.04	−0.5
	O10 (10)	N	HIS 63	A	H-acceptor	3.08	−0.6
	6-ring	ND2	ASN 65	A	pi-H	3.65	−0.5
5	O10 (10)	CB	PRO 62	B	H-acceptor	3.08	−0.5
	O10 (10)	N	HIS 63	B	H-acceptor	3.07	−1.0
	6-ring	ND2	ASN 65	B	pi-H	3.61	−0.5
6	O10 (10)	ND2	ASN 65	A	H-acceptor	2.80	−5.1
	O12 (12)	N	HIS 63	A	H-acceptor	3.16	−0.8
7	O10 (10)	ND2	ASN 65	B	H-acceptor	2.80	−5.1
	O12 (12)	N	HIS 63	B	H-acceptor	3.15	−0.8
8	6-ring	ND2	ASN 65	B	pi-H	3.62	−0.8
9	C11 (11)	O	GLN 15	A	H-donor	3.35	−0.8
	O12 (12)	O	GLN 15	A	H-donor	3.68	−0.5
10	O14 (19)	O	LEU 42	A	H-donor	2.75	−3.9
	O12 (12)	NZ	LYS 122	A	H-acceptor	3.23	−1.2
	6-ring	CG2	THR 39	A	pi-H	3.93	−0.5

**Table 7:** Ligand 38 interactions with wt+wt dimer: the models interacting with both monomers (chain A and B) are highlighted in blue.

Iteration 1							
Model	Atom Ligand (ID)	Atom Receptor	Res. Receptor	Chain (Monomer)	Interaction Type	Distance (Å)	E (kcal/mol)
1	O27 (20)	NZ	LYS 122	A	H-acceptor	3.17	−1.5
	6-ring	CA	ASP 125	A	pi-H	3.64	−0.6
2	O26 (31)	O	GLU 40	B	H-donor	2.89	−1.4
	O27 (20)	NZ	LYS 122	B	H-acceptor	3.19	−1.1
3	O27 (20)	CA	PRO 66	A	H-acceptor	3.57	−0.5
	O24 (29)	NH2	ARG 115	A	H-acceptor	3.10	−1.4
4	—	—	—	—	—	—	—
5	C25 (30)	O	ASP 109	B	H-donor	3.77	−0.5
	O27 (20)	CA	PRO 66	B	H-acceptor	3.64	−0.5
6	O27 (20)	CA	PRO 62	A	H-acceptor	3.35	−1.1
	6-ring	CG	LYS 136	A	pi-H	3.64	−0.5
7	C7 (7)	OG1	THR 137	B	H-donor	3.52	−0.6
	6-ring	CA	GLY 141	B	pi-H	3.78	−0.5
8	O6 (6)	NE1	TRP 32	B	H-acceptor	3.07	−1.8
9	—	—	—	—	—	—	—
10	O24 (29)	ND2	ASN 65	A	H-acceptor	3.02	−1.5
**Iteration 2**							
**Model**	**Atom Ligand (ID)**	**Atom Receptor**	**Res. Receptor**	**Chain (monomer)**	**Interaction type**	**Distance (Å)**	**E (kcal/mol)**
1	O26 (31)	NH1	ARG 141	A	H-donor	3.10	−1.3
	6-ring	CG	ASP 145	A	pi-H	3.50	−0.7
2	O27 (20)	OD1	ASP 108	B	H-acceptor	3.00	−1.5
	C7 (7)	OG1	THR 132	A	H-donor	3.45	−0.6
3	O6 (6)	NE	ARG 115	A	H-acceptor	3.20	−1.2
	6-ring	CA	PRO 66	B	pi-H	3.55	−0.5
4	—	—	—	—	—	—	—
5	C25 (30)	OE1	GLU 124	A	H-donor	3.60	−0.7
	O27 (20)	CA	PRO 62	B	H-acceptor	3.33	−1.0
6	O26 (31)	OG	SER 139	A	H-donor	3.15	−1.3
	6-ring	CB	LYS 122	B	pi-H	3.70	−0.5
7	C7 (7)	OG1	THR 137	B	H-donor	3.40	−0.8
	6-ring	CA	GLY 141	A	pi-H	3.77	−0.6
8	O24 (29)	ND2	ASN 65	B	H-acceptor	3.09	−1.6
9	—	—	—	—	—	—	—
10	O27 (20)	NZ	LYS 122	A	H-acceptor	3.11	−1.5
**Iteration 3**							
**Model**	**Atom Ligand (ID)**	**Atom Receptor**	**Res. Receptor**	**Chain (monomer)**	**Interaction type**	**Distance (Å)**	**E (kcal/mol)**
1	O6 (6)	ND1	HIS 46	A	H-acceptor	3.25	−1.4
	6-ring	CG	ASP 125	B	pi-H	3.60	−0.5
2	O27 (20)	NZ	LYS 136	B	H-acceptor	3.12	−1.3
	C7 (7)	OG1	THR 137	A	H-donor	3.49	−0.6
3	O26 (31)	NE	ARG 115	A	H-acceptor	3.18	−1.2
	6-ring	CA	PRO 66	B	pi-H	3.50	−0.5
4	—	—	—	—	—	—	—
5	C25 (30)	OE2	GLU 40	B	H-donor	3.59	−0.6
	O24 (29)	CA	PRO 62	A	H-acceptor	3.32	−0.9
6	O27 (20)	CA	PRO 66	A	H-acceptor	3.40	−1.1
	6-ring	CG	LYS 136	A	pi-H	3.66	−0.4
7	C7 (7)	OG1	THR 137	A	H-donor	3.41	−0.7
	6-ring	CA	GLY 141	B	pi-H	3.76	−0.6
8	O26 (31)	NE1	TRP 32	B	H-acceptor	3.06	−1.9
9	—	—	—	—	—	—	—
10	O27 (20)	ND2	ASN 65	A	H-acceptor	3.01	−1.4
**Iteration 4**							
**Model**	**Atom Ligand (ID)**	**Atom Receptor**	**Res. Receptor**	**Chain (monomer)**	**Interaction type**	**Distance (Å)**	**E (kcal/mol)**
1	O4 (4)	CA	SER 68	A	H-acceptor	3.74	−0.5
2	O6 (20)	OE2	GLU 132	B	H-donor	3.09	−1.9
3	O6 (20)	O	ARG 69	B	H-donor	3.18	−1.0
4	—	—	—	—	—	—	—
5	—	—	—	—	—	—	—
6	—	—	—	—	—	—	—
7	—	—	—	—	—	—	—
8	O6 (20)	OG1	THR 39	B	H-acceptor	2.73	−0.6
9	—	—	—	—	—	—	—
10	—	—	—	—	—	—	—
**Iteration 5**							
**Model**	**Atom Ligand (ID)**	**Atom Receptor**	**Res. Receptor**	**Chain (monomer)**	**Interaction type**	**Distance (Å)**	**E (kcal/mol)**
1	O4 (4)	CA	SER 68	A	H-acceptor	3.66	−0.5
2	O7 (22)	OE2	GLU 132	B	H-donor	2.70	−4.5
3	O7 (22)	O	ARG 69	A	H-donor	3.01	−1.4
4	—	—	—	—	—	—	—
5	O6 (20)	O	THR 135	B	H-donor	2.93	−1.9
6	6-ring	NZ	LYS 9	A	pi-cation	3.83	−1.1
7	O6 (20)	O	GLU 121	B	H-donor	3.03	−1.6
	O5 (24)	O	ALA 123	B	H-donor	3.17	−0.9
8	—	—	—	—	—	—	—
9	O6 (20)	5-ring	HIS 80	B	H-pi	4.12	−3.4
10	—	—	—	—	—	—	—
**Iteration 6**							
**Model**	**Atom Ligand (ID)**	**Atom Receptor**	**Res. Receptor**	**Chain (monomer)**	**Interaction type**	**Distance (Å)**	**E (kcal/mol)**
1	—	—	—	—	—	—	—
2	O4 (4)	CA	SER 68	A	H-acceptor	3.63	−0.5
3	O7 (22)	OE2	GLU 132	B	H-donor	2.70	−2.9
4	O6 (20)	NE2	HIS 80	B	H-acceptor	3.04	−0.6
5	—	—	—	—	—	—	—
6	O7 (22)	O	ARG 69	A	H-donor	2.94	−1.8
7	—	—	—	—	—	—	—
8	6-ring	NZ	LYS 9	A	pi-cation	3.83	−1.1
9	O6 (20)	O	LEU 84	B	H-donor	3.02	−1.9
10	—	—	—	—	—	—	—
**Iteration 7**							
**Model**	**Atom Ligand (ID)**	**Atom Receptor**	**Res. Receptor**	**Chain (monomer)**	**Interaction type**	**Distance (Å)**	**E (kcal/mol)**
1	O4 (4)	CA	SER 68	A	H-acceptor	3.67	−0.5
2	—	—	—	—	—	—	—
3	O6 (20)	O	THR 135	B	H-donor	2.70	−1.4
4	O6 (20)	O	THR 135	A	H-donor	2.75	−0.7
5	—	—	—	—	—	—	—
6	O6 (20)	O	LEU 84	A	H-donor	3.06	−0.8
7	O5 (24)	O	SER 34	A	H-donor	2.89	−0.8
	6-ring	NZ	LYS 9	A	pi-cation	3.83	−1.1
8	—	—	—	—	—	—	—
9	O6 (20)	O	LEU 84	B	H-donor	3.02	−2.1
10	O5 (24)	O	ASN 53	B	H-donor	2.99	−0.9
	6-ring	NZ	LYS 9	A	pi-cation	3.79	−0.7
**Iteration 8**							
**Model**	**Atom Ligand (ID)**	**Atom Receptor**	**Res. Receptor**	**Chain (monomer)**	**Interaction type**	**Distance (Å)**	**E (kcal/mol)**
1	O4 (4)	CA	SER 68	A	H-acceptor	3.64	−0.5
2	O6 (20)	OE2	GLU 132	B	H-donor	3.10	−3.9
3	O6 (20)	O	ARG 69	B	H-donor	3.21	−1.2
	O7 (22)	O	ARG 69	B	H-donor	2.98	−1.5
4	O6 (20)	O	ARG 69	A	H-donor	3.25	−1.3
5	O5 (24)	O	ALA 123	A	H-donor	3.16	−0.8
6	O5 (24)	O	PRO 74	A	H-donor	3.20	−1.2
7	O7 (22)	O	ASN 86	B	H-donor	2.75	−3.1
8	O5 (24)	O	SER 34	A	H-donor	2.89	−1.4
	6-ring	NZ	LYS 9	A	pi-cation	3.83	−1.1
9	O6 (20)	O	GLN 15	A	H-donor	2.70	−1.1
	6-ring	NZ	LYS 9	A	pi-cation	3.80	−0.7
10	—	—	—	—	—	—	—
**Iteration 9**							
**Model**	**Atom Ligand (ID)**	**Atom Receptor**	**Res. Receptor**	**Chain (monomer)**	**Interaction type**	**Distance (Å)**	**E (kcal/mol)**
1	O6 (20)	OE2	GLU 132	A	H-donor	3.16	−1.3
	O4 (4)	CA	SER 68	A	H-acceptor	3.64	−0.5
2	—	—	—	—	—	—	—
3	—	—	—	—	—	—	—
4	O6 (20)	O	THR 135	A	H-donor	2.74	−0.9
5	O6 (20)	O	GLU 121	A	H-donor	3.00	−2.0
	O5 (24)	O	ALA 123	A	H-donor	3.15	−1.4
6	O6 (20)	O	LEU 84	A	H-donor	3.05	−0.9
	O7 (22)	O	ASN 86	A	H-donor	2.70	−2.4
	O5 (24)	O	PRO 74	A	H-donor	3.18	−1.0
7	O6 (20)	O	LEU 84	B	H-donor	3.02	−1.8
8	O5 (24)	O	GLN 15	A	H-donor	2.75	−1.4
	6-ring	NZ	LYS 9	A	pi-cation	3.83	−1.1
9	O7 22	O	GLU 40	B	H-donor	2.86	−1.5
10	O6 20	O	SER 34	A	H-donor	2.97	−1.4
	O5 24	O	ASN 53	B	H-donor	2.99	−0.5
	6-ring	NZ	LYS 9	A	pi-cation	3.79	−0.7
**Iteration 10**							
**Model**	**Atom Ligand (ID)**	**Atom Receptor**	**Res. Receptor**	**Chain (monomer)**	**Interaction type**	**Distance (Å)**	**E (kcal/mol)**
1	O4 (4)	CA	SER 68	A	H-acceptor	3.63	−0.5
2	—	—	—	—	—	—	—
3	—	—	—	—	—	—	—
4	O6 (20)	O	THR 135	A	H-donor	2.75	−3.0
	O6 (20)	CA	LYS 70	A	H-acceptor	3.33	−0.6
5	—	—	—	—	—	—	—
6	O6 (20)	O	ASN 86	A	H-donor	2.95	−2.2
	O5 (24)	O	PRO 74	A	H-donor	3.19	−1.3
7	6-ring	NZ	LYS 9	A	pi-cation	3.82	−1.1
8	O5 (24)	O	ALA 123	B	H-donor	3.13	−1.1
9	—	—	—	—	—	—	—
10	6-ring	NZ	LYS 9	A	pi-cation	3.79	−0.7

**Table 8:** Compound library.

No.	Ligand Name	Molecular Mass (g/mol)	LogP	H-Bond Donors	H-Bond Acceptors	Lipinski Rule of Five Compliance
1	1-(2,5-dideoxy-5-pyrrolidin-1-yl-β-L-erythro-pentofuranosyl)uracil	260.00	1.5	2	5	Yes
2	2′,3′-Dideoxythymidine-5′-Monophosphate	306.21	−1.5	3	7	Yes
3	2′,5′-Dideoxyuridine	228.00	−1.0	2	5	Yes
4	2′-Deoxy-5-(hydroxymethyl)uridine 5′-(dihydrogen phosphate)	338.21	−2.0	3	8	Yes
5	2′-Deoxyuridine	228.20	−1.0	2	5	Yes
6	5′-Deoxy-5′-piperidin-1-ylthymidine	270.00	1.0	2	5	Yes
7	5-Methyl-2′-fluoroauracil F-18	150.00	0.5	1	4	Yes
8	Alovudine F-18	250.00	1.0	2	5	Yes
9	Alovudine	250.00	1.0	2	5	Yes
10	Brivudine Monophosphate	413.11	0.5	4	8	Yes
11	Brivudine	333.13	1.0	3	6	Yes
12	Broxuridine	306.00	0.5	2	7	Yes
13	Clevudine	272.00	1.5	2	5	Yes
14	Ebselen	274.98	3.13	0	0	Yes
15	Edoxudine	306.00	0.5	2	7	Yes
16	Epigallocatechin Gallate	458.00	1.2	8	11	No
17	Ethaselen	300.00	2.0	1	4	Yes
18	Ethylarabinosyluracil	260.00	1.0	2	5	Yes
19	Floxuridine	264.00	−1.0	2	6	Yes
20	Idoxuridine	354.00	0.5	2	7	Yes
21	Netivudine	272.00	1.5	2	5	Yes
22	PRG_A01	300.00	1.0	2	6	Yes
23	Rotenone	394.00	4.0	0	5	Yes
24	S_XL6	320.00	2.0	2	6	Yes
25	Sorivudine	316.00	1.0	2	6	Yes
26	Telbivudine	258.00	1.5	2	5	Yes
27	Thymidine Monophosphate	322.00	−1.5	3	8	Yes
28	Thymidine 3′,5′-Diphosphate	402.00	−2.5	4	10	Yes
29	Thymidine 5′-Diphosphate	402.00	−2.5	4	10	Yes
30	Thymidine 5′-(dithio)phosphate	370.00	−2.0	3	9	Yes
31	Thymidine 5′-Thiophosphate	354.00	−2.0	3	8	Yes
32	TMP (Thymidine Monophosphate)	322.00	−1.5	3	8	Yes
33	Trifluridine	296.00	0.5	2	7	Yes
34	Uridine	244.00	−2.0	3	6	Yes
35	Zidovudine	267.00	1.0	2	6	Yes
36	Baicalein	270.24	2.5	3	5	Yes
37	Lovastatin	404.54	4.57	1	5	Yes
38	Quercetin	302.24	1.5	5	7	Yes
39	Simvastatin	418.57	4.68	1	5	Yes

**Table 9:** Best affinity ligands—structural formula.

Ligand Number	Ligand Name	Structural Formula
22	PRG-A01	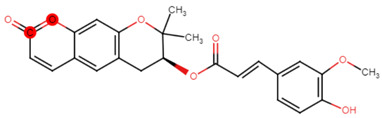
36	Baicalein	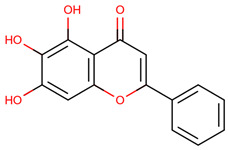
38	Quercetin	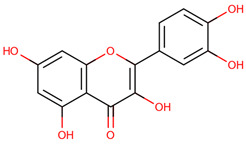

**Table 10:** Best larger cluster-forming ligands—structural formula.

Ligand Number	Ligand Name	Structural Formula
34	Uridine	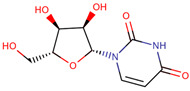
37	Lovastatin	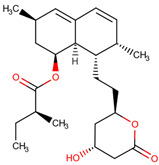
24	S-XL6	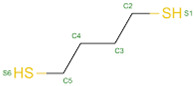

## Data Availability

The data generated in this study are available upon request.

## Supplementary Materials

The following supporting information can be downloaded at https://www.mdpi.com/article/10.3390/ijms26104660/s1.
