# Supplementary material for: Computational Search for Inhibitors of SOD1 Mutant Infectivity as Potential Therapeutics for ALS Disease
Source: Int J Mol Sci. 2025 May 13;26(10):4660. doi: 10.3390/ijms26104660 (PMC12111112; doi:10.3390/ijms26104660)
Supplement: Supplementary file 1 [file ijms-26-04660-s001.zip › ijms-3559923-supplementary.pdf]

Supplementary information for

"Docking results
Binding energies and spatial pose clustering results"

Binding energies for each ligand and dimer

The following graphs show the binding or energetic affinity scores calculated by Vina for each ligand with one of the eight selected dimers. The X-axis reports the ligands, numbered progressively, while the Y-axis represents the Vina scores in kcal/mol, where more negative values indicate a higher binding affinity. Purple dots correspond to the individual Vina scores obtained for each pose, orange stars represent the average score for each ligand, while red diamonds highlight the five ligands with the most negative average score, meaning those with the highest binding affinity. Each ligand has a distribution of scores since multiple docking attempts were performed: ten executions of the Vina program with the same parameters, each generating ten poses, resulting in a total of 100 poses per ligand. Some ligands show greater dispersion in their scores, suggesting variability in their binding affinity. The five ligands with the best scores are clearly identified in red. The Y-axis scale ranges approximately from -9 to -3 kcal/mol, consistent with typical binding energy values in molecular docking studies.

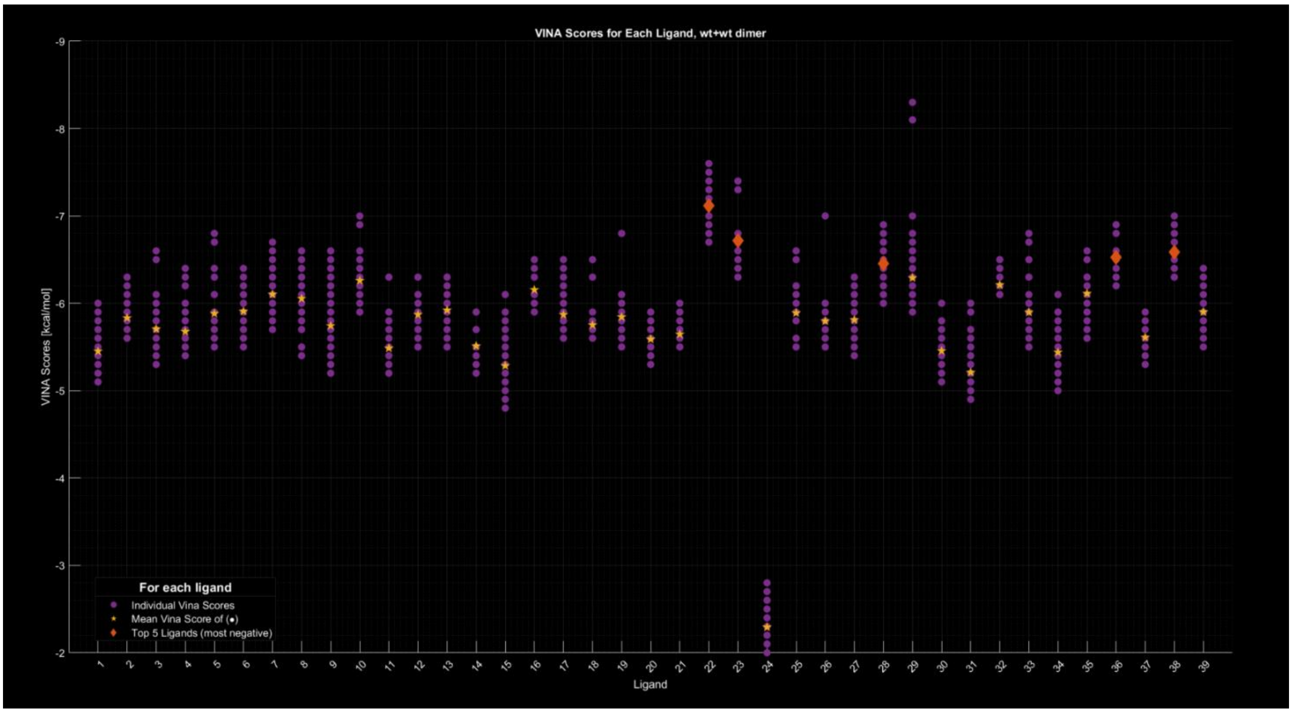
VINA Scores for Each Ligand, wt+wt dimer
VINA Scores [kcal/mol]
Ligand
For each ligand
Individual Vina Scores
Mean Vina Score of (●)
Top 5 Ligands (most negative)

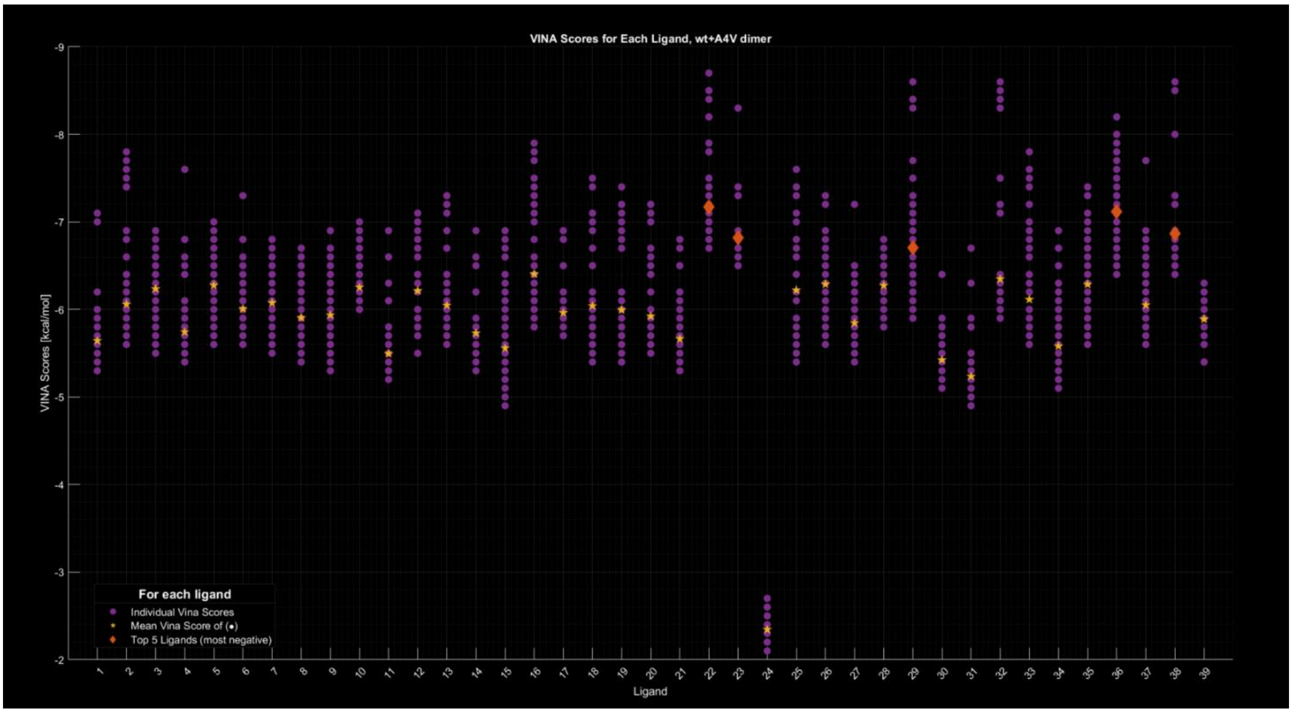
VINA Scores for Each Ligand, wt+A4V dimer
VINA Scores [kcal/mol]
Ligand
For each ligand
Individual Vina Scores
Mean Vina Score of (●)
Top 5 Ligands (most negative)

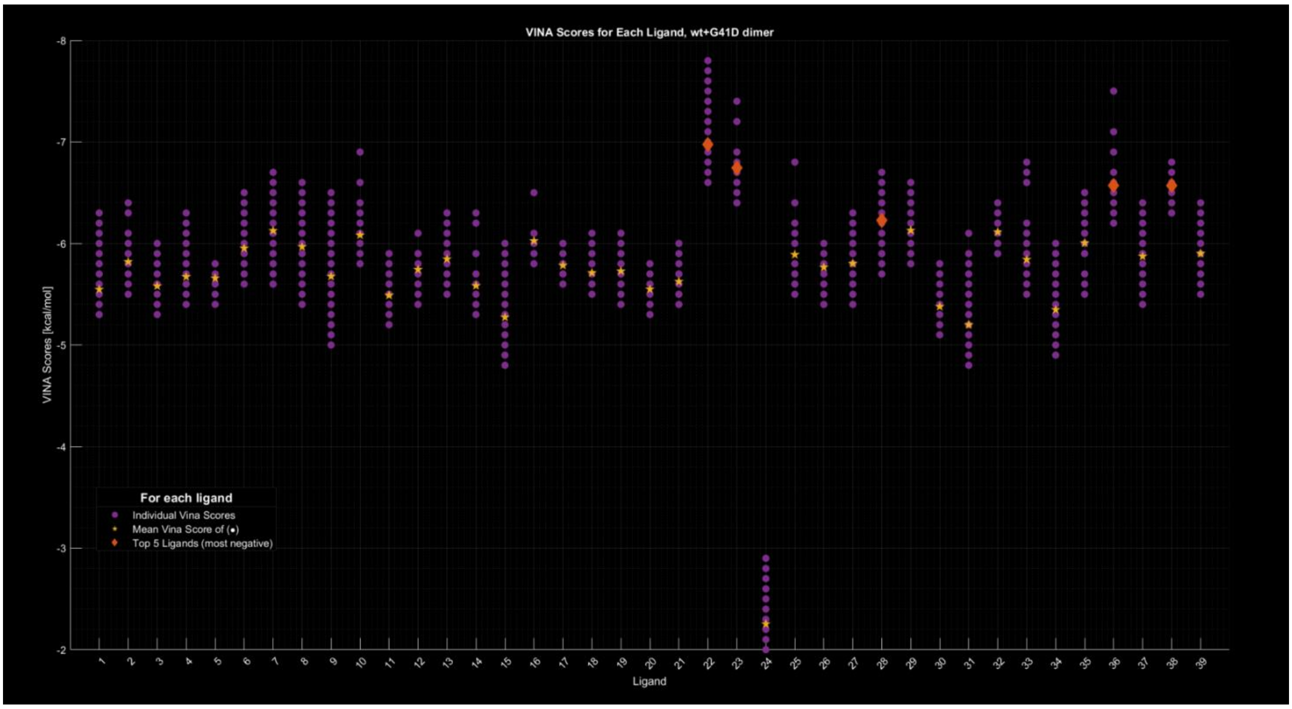
VINA Scores for Each Ligand, wt+G41D dimer
VINA Scores [kcal/mol]
Ligand
For each ligand
Individual Vina Scores
Mean Vina Score of (●)
Top 5 Ligands (most negative)

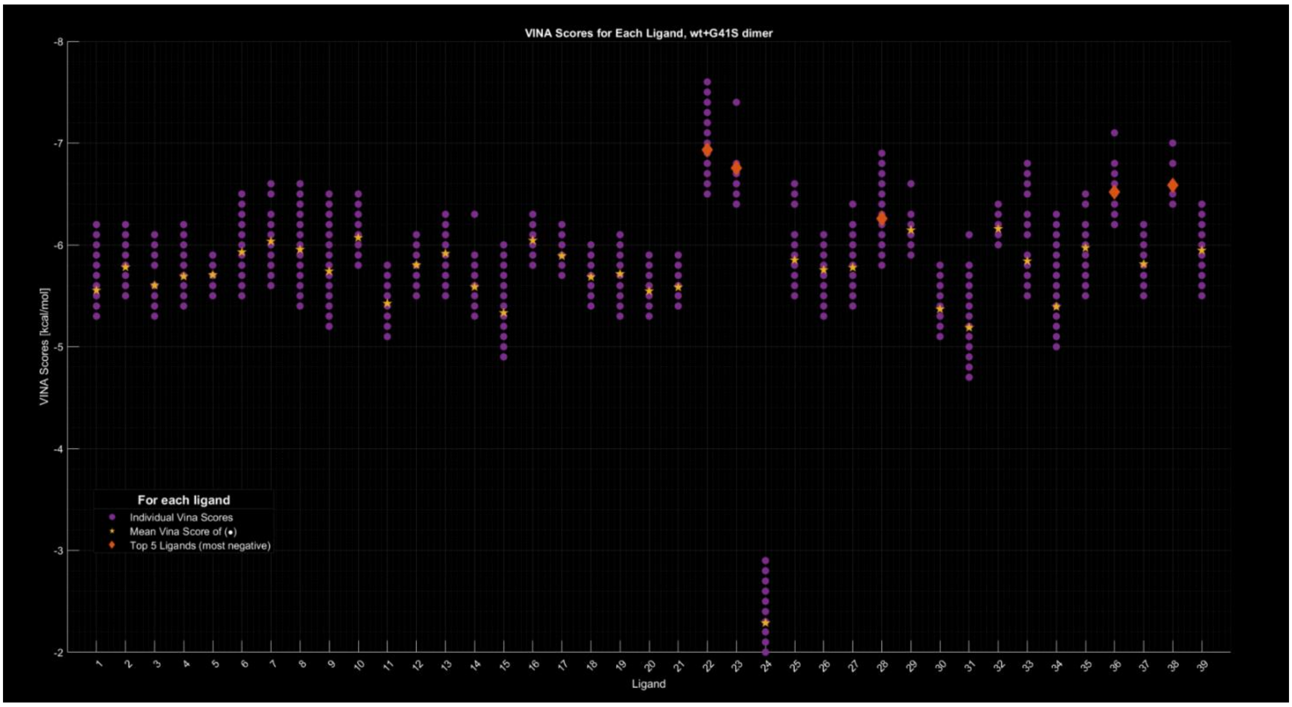
VINA Scores for Each Ligand, wt+G41S dimer
VINA Scores [kcal/mol]
Ligand
For each ligand
Individual Vina Scores
Mean Vina Score of (●)
Top 5 Ligands (most negative)

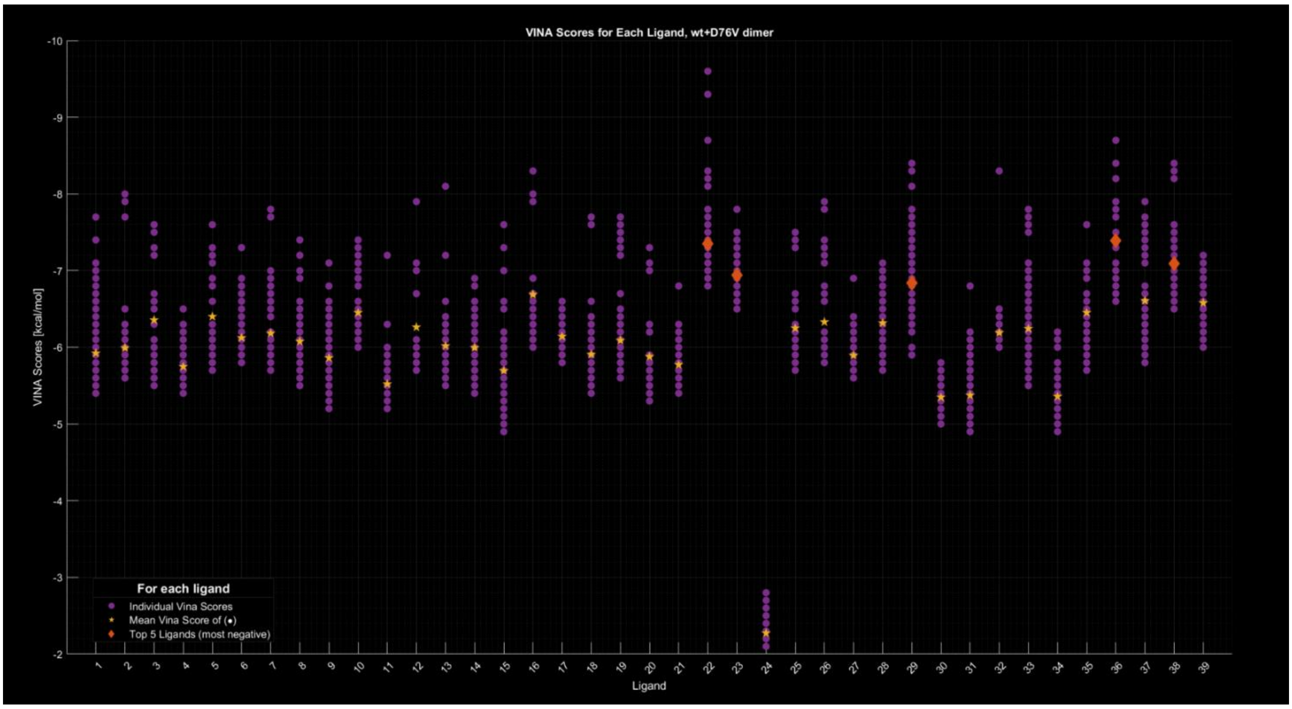
VINA Scores for Each Ligand, wt+D76V dimer
VINA Scores [kcal/mol]
Ligand
For each ligand
Individual Vina Scores
Mean Vina Score of (●)
Top 5 Ligands (most negative)

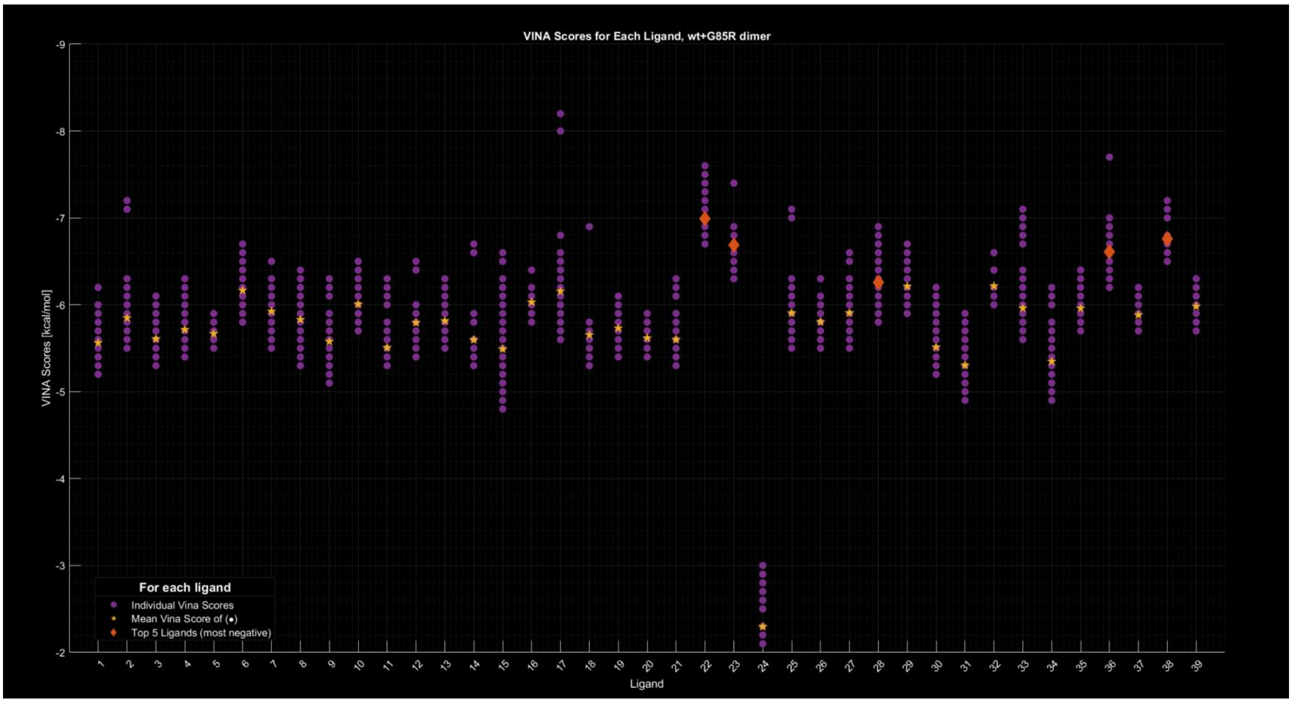
VINA Scores for Each Ligand, wt+G85R dimer
VINA Scores [kcal/mol]
Ligand
For each ligand
Individual Vina Scores
Mean Vina Score of (●)
Top 5 Ligands (most negative)

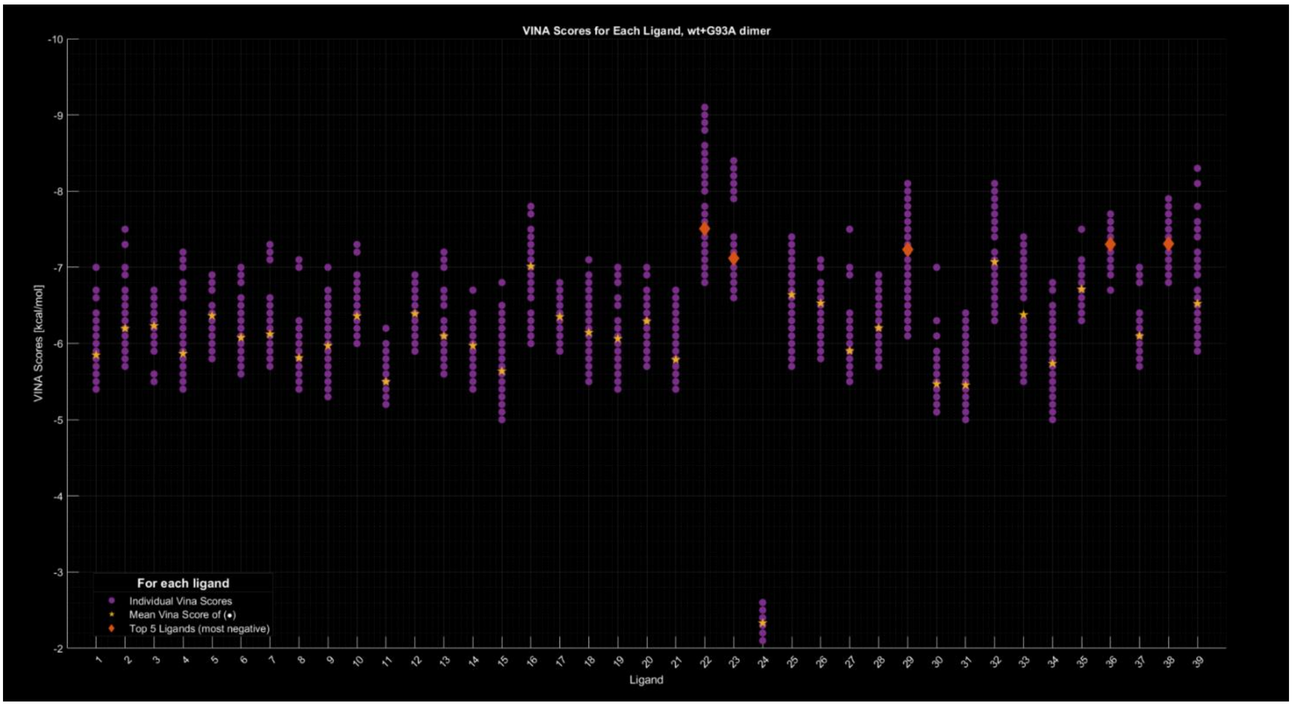
VINA Scores for Each Ligand, wt+G93A dimer
VINA Scores [kcal/mol]
Ligand
For each ligand
Individual Vina Scores
Mean Vina Score of (•)
Top 5 Ligands (most negative)

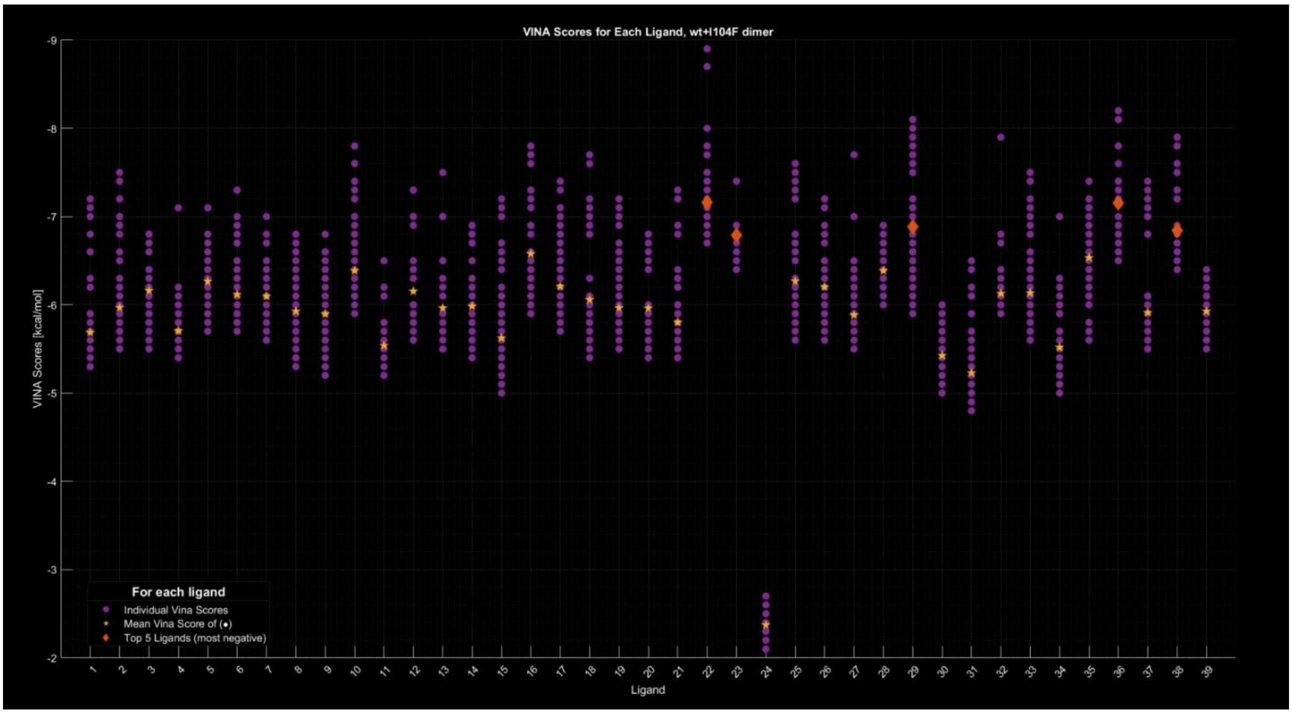
VINA Scores for Each Ligand, wt+I104F dimer
VINA Scores [kcal/mol]
Ligand
For each ligand
Individual Vina Scores
Mean Vina Score of (•)
Top 5 Ligands (most negative)

Pose clusters for each ligand and dimer

The graphs represent the cluster sizes for each ligand in a molecular docking experiment with various dimers. The X-axis displays the ligands, numbered progressively, while the Y-axis represents the cluster sizes, expressed as the number of poses per cluster. Blue dots indicate the cluster sizes for each ligand, while red dots highlight the five ligands with the largest cluster, meaning those with the highest number of grouped poses. The graphs show a highly variable distribution, with some ligands forming larger clusters than others, suggesting greater conformational stability in their docking poses.

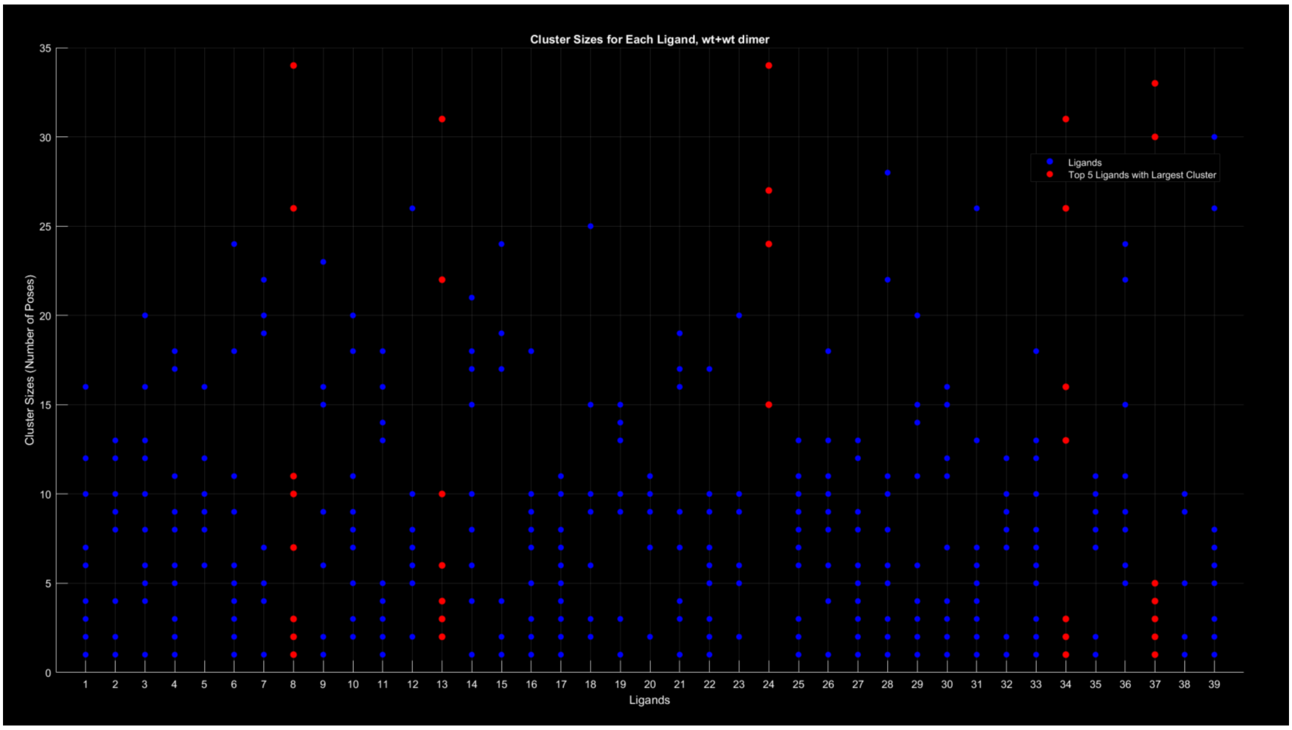

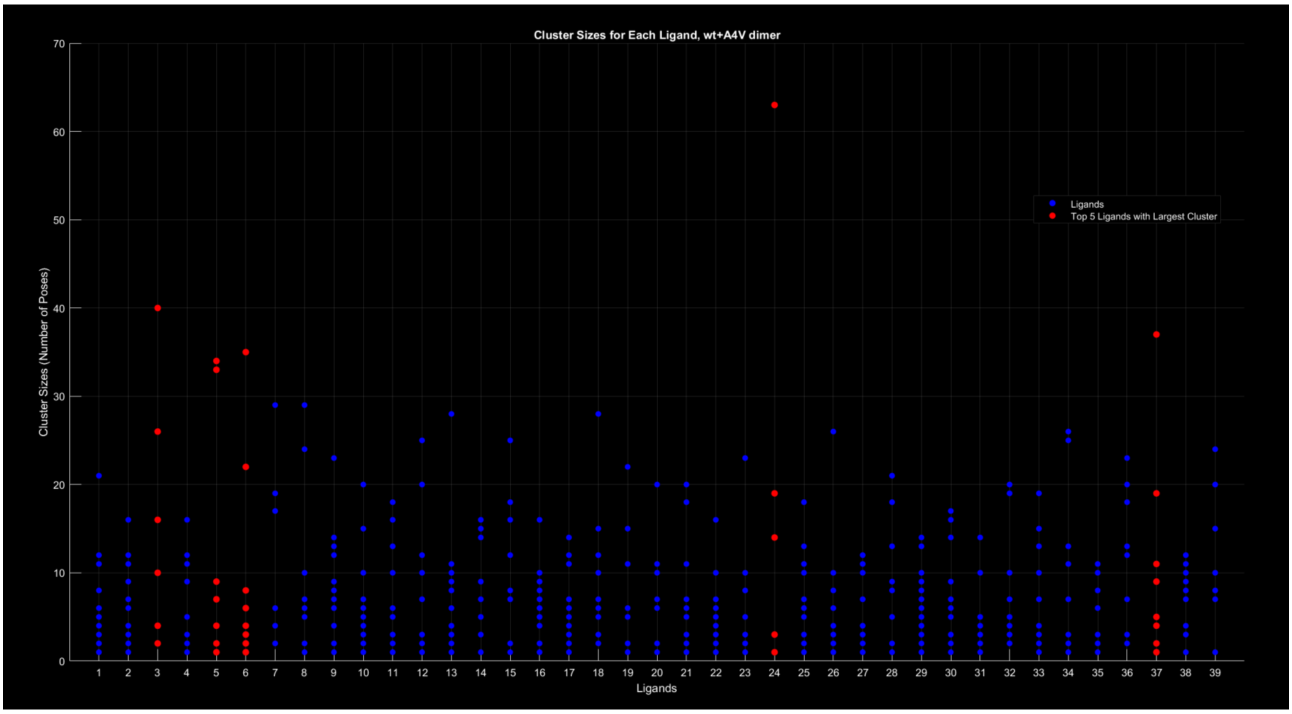

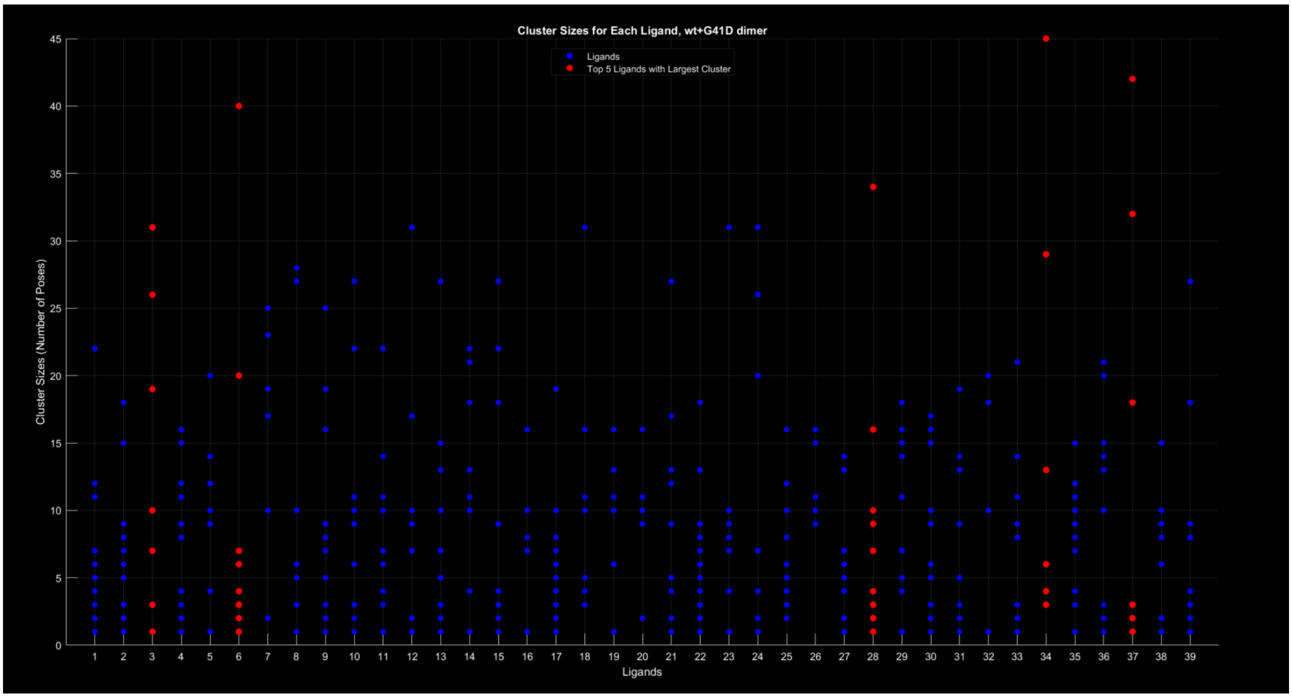
Cluster Sizes for Each Ligand, wt+G41D dimer
Ligands
Top 5 Ligands with Largest Cluster
Cluster Sizes (Number of Poses)
Ligands

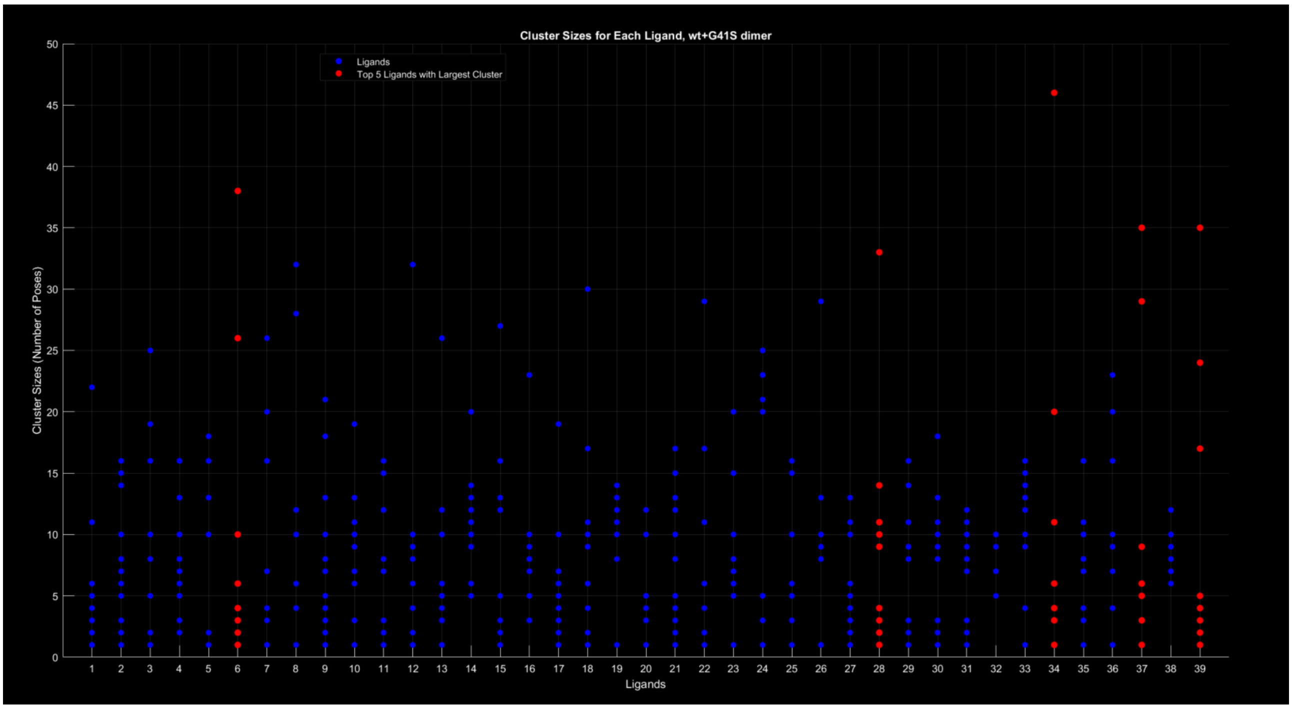
Cluster Sizes for Each Ligand, wt+G41S dimer
Ligands
Top 5 Ligands with Largest Cluster
Cluster Sizes (Number of Poses)
Ligands

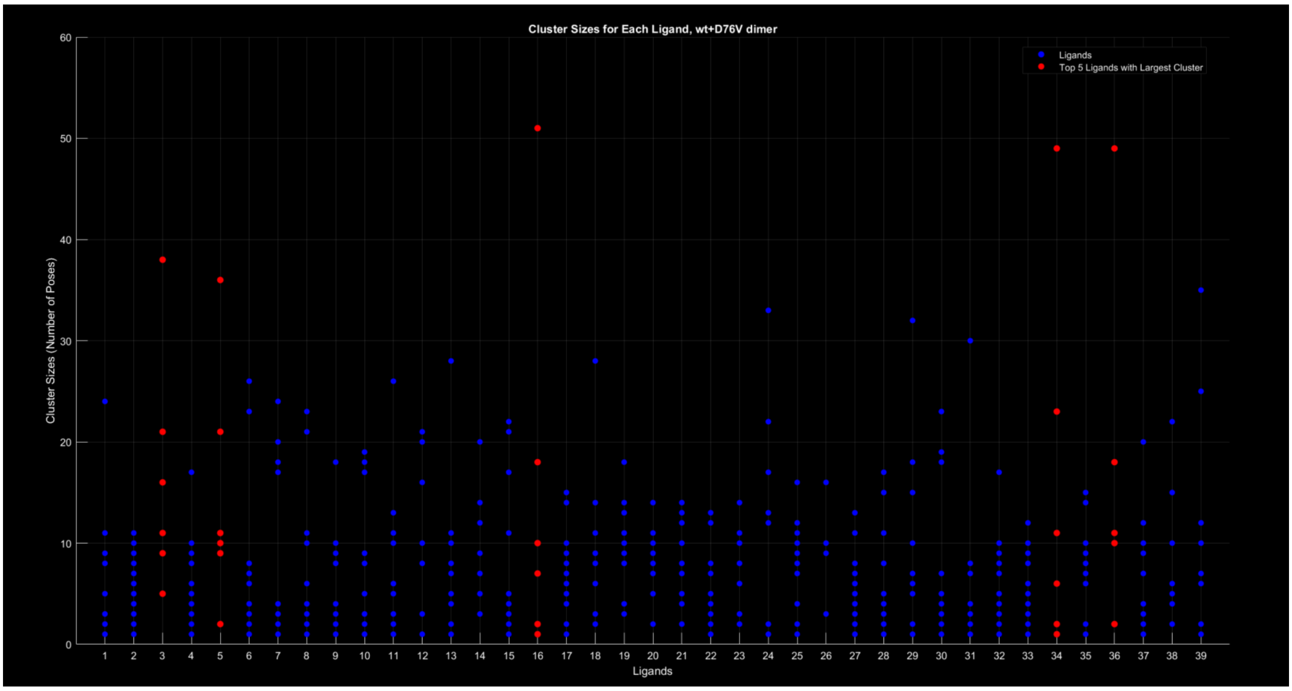
Cluster Sizes for Each Ligand, wt+D76V dimer
Ligands
Top 5 Ligands with Largest Cluster
Cluster Sizes (Number of Poses)
Ligands

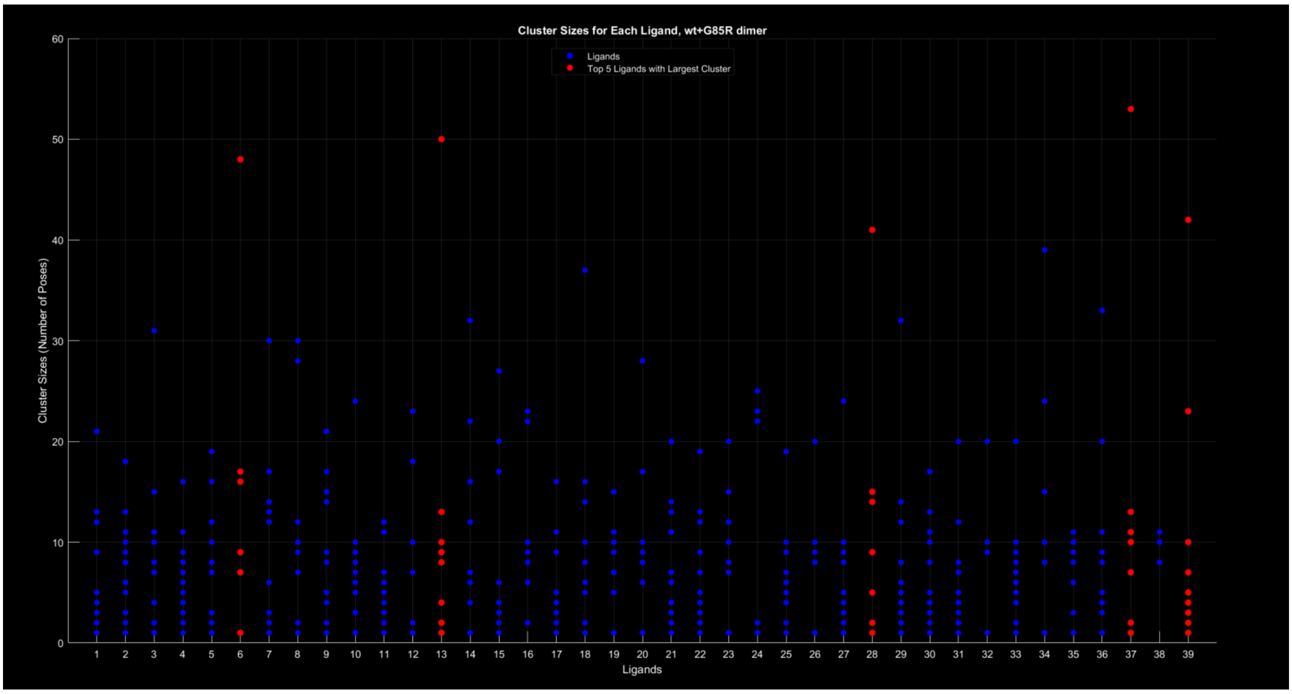
Cluster Sizes for Each Ligand, wt+G85R dimer
Ligands
Top 5 Ligands with Largest Cluster
Cluster Sizes (Number of Poses)
Ligands

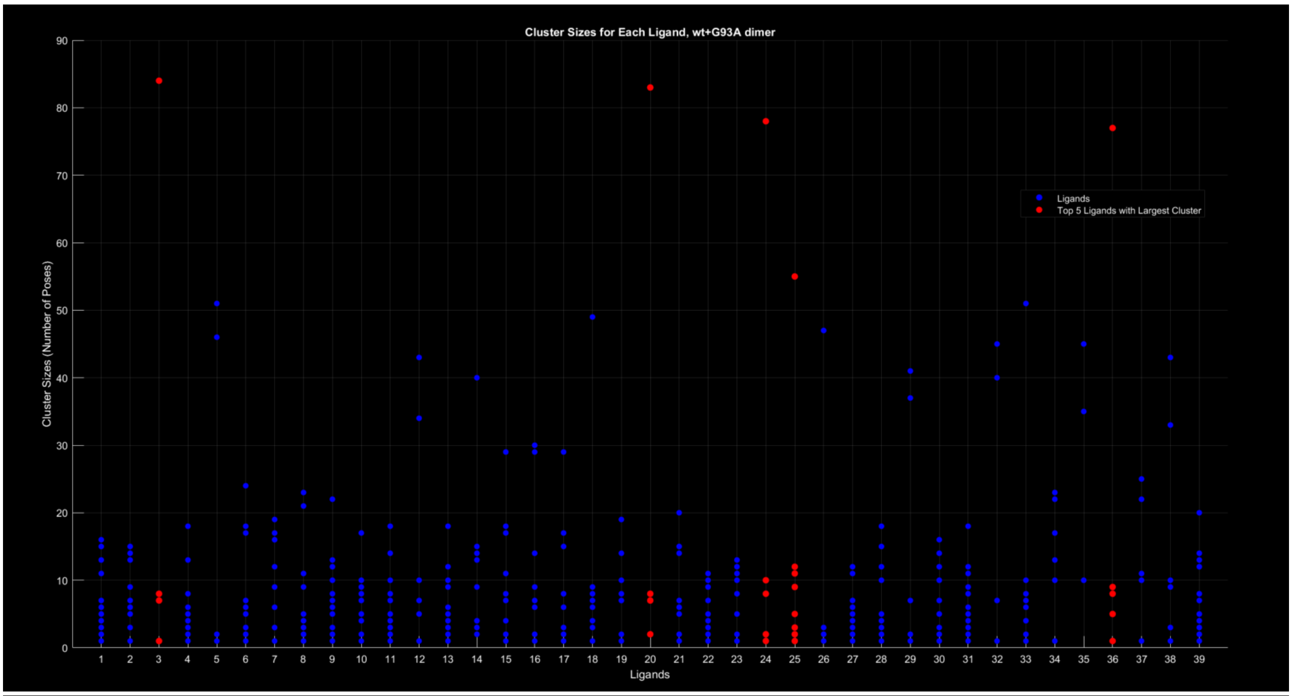
Cluster Sizes for Each Ligand, wt+G93A dimer
Ligands
Top 5 Ligands with Largest Cluster
Cluster Sizes (Number of Poses)
Ligands

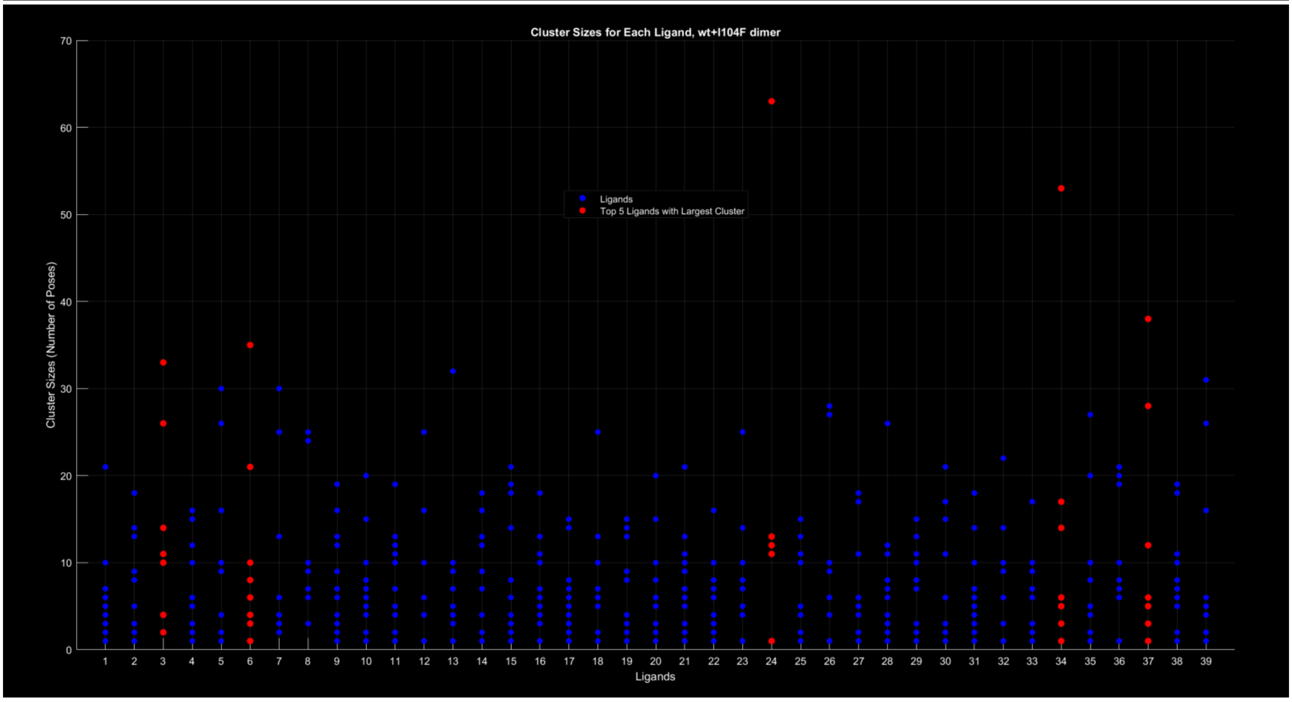
Cluster Sizes for Each Ligand, wt+I104F dimer
Ligands
Top 5 Ligands with Largest Cluster
Cluster Sizes (Number of Poses)
Ligands
